# Laser and anti-vascular endothelial growth factor treatment for drusenoid pigment epithelial detachment in age-related macular degeneration

**DOI:** 10.1038/s41598-020-71401-3

**Published:** 2020-09-01

**Authors:** Min Seok Kim, Na-Kyung Ryoo, Kyu Hyung Park

**Affiliations:** 1 Department of Ophthalmology, Seoul National University College of Medicine, Seoul, South Korea; 2 Department of Ophthalmology, Seoul National University Bundang Hospital, 173-82 Gumi-ro, Bundang-gu, Seongnam-si, Gyeonggi-do 13620 South Korea; 3 Department of Ophthalmology, Veterans Health Service Medical Center, Seoul, South Korea

**Keywords:** Macular degeneration, Retinal diseases

## Abstract

This study aims to report the 12 months results of efficacy and safety of laser photocoagulation and anti-vascular endothelial growth factor (VEGF) injections for drusenoid pigment epithelial detachment (dPED). In this prospective study, patients with treatment naïve bilateral intermediate age-related macular degeneration, featuring dPED, with visual acuity ≤ 83 letters were enrolled. The study group received PASCAL laser (532 nm) along the periphery of the dPED, and the fellow eye served as a control group. To prevent complications of choroidal neovascularization, intravitreal anti-VEGF injections to laser treated eye were performed on a 3-month interval up to 1 year. Primary outcomes—drusen area, PED height—and secondary outcomes—best-corrected visual acuity (BCVA), contrast sensitivity, degree of metamorphopsia, NEI-VFQ 25, and fundus autofluorescence—were analyzed. Among 21 patients, a total of 20 patients satisfied the 12 months follow-up. Drusen area and PED height decreased significantly in the laser group, while no significant change appeared in the control group (74.1% vs. − 3.5%, P < 0.001; 76.6% vs. 0.1%, P < 0.001). Mean BCVA improved 4.6 letters in the laser group (vs. 1.1 letters in the control group, P = 0.019). As for safety, one study eye developed retinal pigment epithelial tear, and one control eye developed retinal angiomatous proliferation. Low energy laser photocoagulation and anti-VEGF injection in eyes with dPED showed some improvement in visual acuity. dPED regressed without developing center involving GA in the study eye, but a longer term follow-up is necessary to reveal the efficacy and safety of these treatments. The 2-year results of this study will be followed to reveal long term efficacy and safety of the treatment for dPED.

## Introduction

Drusen—amorphous yellowish deposits beneath the sensory retina—are a predisposing risk factor for development of age-related macular degeneration (AMD)^1^. Several randomized clinical trials of laser treatment for drusen, with the hope to slow progression to late AMD, were conducted in the 1990s and early 2000s^2–6^. A meta-analysis of these trials implied that laser application (argon, krypton, dye or diode etc.) may lead to reduction of drusen to a certain extent. Unfortunately, the risk of developing choroidal neovascularization (CNV), geographic atrophy (GA) or visual acuity loss was not altered, which was considered partially attributable to the non-selective delivery of laser energy causing collateral thermal damage to the surrounding retinal tissues^7^. However, recent sophistication and advancements in laser applications, i.e., subthreshold nanosecond laser, micropulse laser etc., have opened up a new opportunity of retinal laser therapies and re-initiated trials in the treatment of non-exudative AMD^8,9^.

In previous studies, the efficacy and safety of laser treatment for intermediate AMD have mainly focused on slowing progression to late AMD and the response of soft drusen. However, response of drusenoid pigment epithelial detachment (PED), another predisposing factor of AMD progression, to laser treatment has not been established^10–12^. Drusenoid PED, first described by Casswell in 1985^13^, is a well-defined, shallow separation of the retinal pigment epithelium (RPE) from Bruch’s membrane in association with large confluent soft drusen and is usually located in the central macula^14^. Together with soft drusen and retinal pigmentary changes, drusenoid PED has been previously identified as a fundus lesion associated with increased risk of progression to advanced AMD^1,11^. In a prospective study, drusenoid PED was characterized by a high rate of progression to both central GA (19%) and neovascular AMD (23%) within 5 years^11^. Decline of visual acuity and progressive pigmentary changes in drusenoid PED were also found, regardless of progression to advanced forms of AMD^11^. Persistent PED can cause GA over time because of long-term separation of the RPE from the underlying choriocapillaris^14,15^. Despite these unfavorable outcomes of drusenoid PED in AMD, no effective treatment has been reported up to date. High-dose statins have been reported to result in drusenoid PED resolution, yet there is little evidence^16^.

Therefore, the aim of this study was to evaluate the functional and anatomic outcomes as well as short-term safety of laser in eyes with drusenoid PED. Unlike prior trials, we used 532 nm Nd:YAG laser at a very short pulse duration and prophylactic intravitreal anti-vascular endothelial growth factor (VEGF) injections to reduce the risk of CNV.

## Methods

A prospective, institutional, interventional non-randomized clinical trial was conducted in patients with bilateral drusenoid PED. This study is registered at clincaltrials.gov (Identifier: NCT02960828; Date of registration: 10/11/2016). The study was conducted according to the International Conference on Harmonization for Good Clinical Practice and the tenets of the Declaration of Helsinki, with the protocol approved by the institutional review board of Seoul National University Bundang Hospital (IRB no. B-1508/312-004). Written informed consent was obtained from all study participants. This clinical trial is an on-going 2-year study, with a 12 months results for efficacy and safety of laser treatment and anti-VEGF injections.

### Study population

Eligible participants were 50 years of age or older, with intermediate AMD, especially treatment-naïve drusenoid PED in both eyes. Drusenoid PED was defined as an elevated mound with confluent soft drusen larger than 500 μm under the macula, viewed on color fundus photography. All participants were required to have a best-corrected visual acuity (BCVA) of ≤ 83 letters using standard Early Treatment Diabetic Retinopathy Study (ETDRS) chart. We prospectively recruited patients who visited Seoul National University Bundang Hospital between January 2016 and May 2018.

Exclusion criteria were any evidence of advanced AMD including GA or CNV at baseline examination on color fundus photography or spectral-domain optical coherence tomography (SD-OCT), past treatment for CNV, patient with only one visually effective eye (i.e., only eye), other macular or retinal diseases other than AMD those would interfere with vision, any intraocular surgery within a year (or cataract surgery within 6 months), any on-going or impending systemic treatment that may have retinal toxicity, or those with fluorescein allergy.

Sample size was pre-determined by McNemar Test Power Analysis. With the power of 80%, a significance level of 0.05, difference 0.45 and a proportion discordant 0.05, the required calculated sample size was 21. Therefore, we aimed to enroll a minimum of 21 participants (42 eyes) to analyze the effect of treatment with sufficient statistical power.

### Ocular examinations

**Figure 1:**
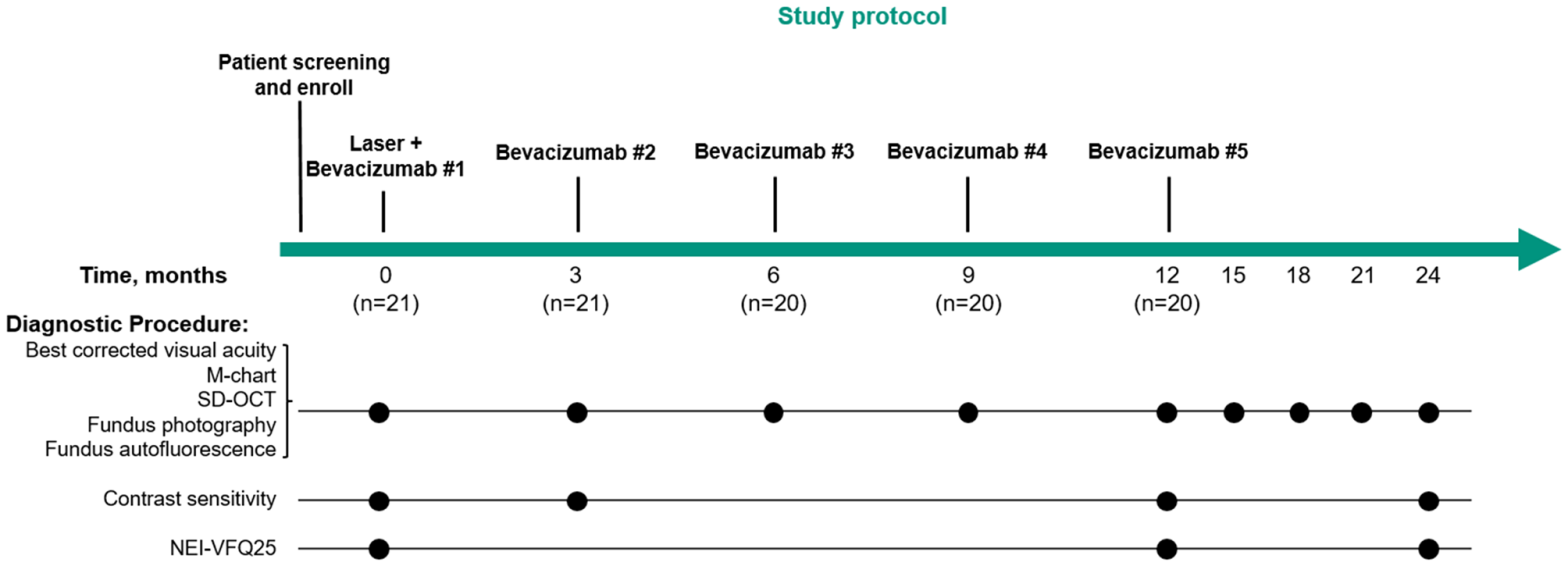
Diagnostic procedures and treatment schedules of the study. SD-OCT = Spectral-domain optical coherence tomography, NEI-VFQ = National Eye Institute Visual Function Questionnaires.

Ocular examinations were done on baseline and follow-up visits, which were scheduled at 3, 6, 9, 12, 15, 18, 21 and 24 months after enrollment (Fig. 1). Baseline assessment included BCVA, intraocular pressure, slit-lamp biomicroscopy, color fundus photography (VX-10; Kowa Optimed, Tokyo, Japan), fluorescein angiography, SD-OCT scans of macula (Spectralis OCT, Heidelberg Engineering, Heidelberg, Germany), fundus autofluorescence (FAF; Spectralis HRA + OCT, Heidelberg Engineering, Heidelberg, Germany), contrast sensitivity (VCTS-6000 chart, Vistech Consultants, Inc., Dayton, OH, USA), M-charts (Inami Co., Tokyo, Japan), and National Eye Institute Visual Function Questionnaires (NEI-VFQ 25). At each scheduled follow-up, BCVA measurement, color fundus photography, SD-OCT, FAF and M-chart were performed. Visual acuity was scored as the number of letters read correctly at 4-m distance. For SD-OCT, over 30 frames were averaged to obtain 1 horizontal and vertical high-resolution image, and 25 B-scans of the central 30° × 20° area (10 frames were averaged for each image) were acquired for each eye, using the automatic scan alignment feature for follow-up scans. The contrast sensitivity was measured at baseline, month 3, and month 12 for five spatial frequencies: 1.5, 3, 6, 12 and 18 cycles/degree. Accompanying validated scoring sheets were used to record contrast sensitivity responses for analysis. The degree of vertical and horizontal metamorphopsia was quantified using M-charts which is comprised of consecutive dotted lines, and the patients have to state whether the lines are distorted or straight^17^. Patients were questioned about Korean version of the NEI-VFQ 25 annually. NEI-VFQ 25, a vision-specific quality of life instrument, comprises 25 items to assess the difficulty of visual symptoms or day-to-day activities with 11 vision-related domains^18^. Fluorescein angiography was performed at baseline and whenever necessary to evaluate symptoms or changes in the fundus and SD-OCT suggested CNV.

### Intervention

The eye with lower vision was assigned to the study group, and the fellow eye served as a control (observation) group. However, if both eyes have same visual acuity, random selection was done for each group. A block designated right or left was placed in a box and a masked investigator blind-draw one of the blocks to determine one eye as a control. Eyes assigned to treatment received photocoagulation using 532 nm Nd: YAG laser (PASCAL, OptiMedica, Santa Clara, CA) with 100 μm spot size, power of 100–175 mW and 20–40 ms’ duration, with the goal of creating a nearly invisible subtle burn. After instillation of a drop of topical anesthesia (0.5% proparacaine hydrochloride), a contact lens (Ocular Mainster High Magnification; Ocular inst, Bellevue, WA, USA) was placed on the cornea. Laser photocoagulation was applied along the periphery of the targeted drusenoid PED and drusen (Supplementary Fig. S1 online). The number of laser shots per eye, depends on the number of large drusen and the extent of drusenoid PED. In the case of a large druse, one shot was applied at the furthest location from the macula on the boundary of the drusen. On a large drusenoid PED, laser was also applied on boundary at the furthest location from the macula with a minimum of 500 μm distance between laser spots. Therefore, more than one shot was applied on a large drusenoid PED. No laser spots were within 500 μm from the fovea center in any case.

Three months after the initial treatment, additional laser treatment was considered if there was no reduction in drusen size at all on the fundus photography. The spot size, duration, and intensity of the laser were maintained the same as the initial treatment.

To prevent the development of CNV, participants received anti-VEGF injections (Bevacizumab, Genentech; Roche, Basil, Switzerland, 1.25 mg/0.05 ml) in the study eye concomitant with baseline laser treatment and four quarterly injections over the next 12 months period. An additional visit was carried out for safety assessment 1 month after each injection.

### Main outcome measures

All baseline and follow-up images were reviewed and graded by two retinal specialists (MSK and NKR), who were masked to whether the eyes were treated or not. When discrepancies arose, the two observers discussed their evaluations and came to an agreement.

Data analysis was performed to evaluate outcomes comparing the study eye to the control eye, and comparing features over time in each arm: (1) primary outcome measures: drusen area on color fundus photography and PED height on SD-OCT, (2) secondary outcome measures: BCVA, contrast sensitivity, degree of metamorphopsia, NEI-VFQ 25, presence of GA or CNV, and FAF findings. Drusen area was measured using ImageJ software (National Institutes of Health, Bethesda, MD, USA) using the ‘freehand selection tool’, and assessed by ratios over time. Only the drusen within the vascular arcades were measured for analysis. PED height was measured from the pigment epithelium layer to Bruch’s membrane at its greatest height on the horizontal section crossing the fovea on SD-OCT. This value was manually measured using built-in software.

All images including the fluorescein angiography, fundus photography, and SD-OCT images, from baseline to 12 months, were also graded for the presence of GA or CNV. GA was defined as a sharply delineated area of RPE hypopigmentation or depigmentation, at least 175 μm in diameter on color fundus photography. We defined the presence of 2 features, the subsidence of the outer plexiform layer and inner nuclear layer and the hyporeflective wedge-shaped band on SD-OCT, as nascent geographic atrophy (nGA), and observed whether nGA and GA occurred during follow up^19^. The development of CNV was considered as having occurred when there was evidence of dye leakage on fluorescein angiography or subretinal fluid, exudation and hemorrhages on fundus photography or neovascular lesion on SD-OCT.

### Statistical analysis

Paired t-test was performed to analyze the changes over time within each arm. Serial changes in outcomes were compared between the two arms using generalized estimating equation. The statistical analysis was performed using SPSS version 25.0 (IBM Corp., Armonk, NY, USA). *P* values < 0.05 were considered to be statistically significant.

## Results

A total of 21 patients were recruited and 20 patients (20 eyes for treatment arm, fellow 20 eyes for control arm) who completed the 12 months of follow-up were included in this analysis. One patient was excluded from the study at month 6 as a result of follow up loss. Mean patient age was 72.3 ± 5.6 years (range, 57 to 81 years) and 2/20 (10%) patients were male. Additional laser treatment was performed in one eye (case 6) because of no reduction in drusen size on fundus photography at month 3. 13 eyes of 7 patients had a history of cataract surgery without any complication at least 1 year before the study enrollment. Table 1 lists the demographic and baseline clinical characteristics of the patients. At baseline, no differences in drusen area and PED height were seen between the 2 eyes (*P* = 0.244, 0.051, respectively). The average BCVA was 70.7 ± 8.9 letters in study eye and 77.1 ± 7.0 letters in control eye (*P* < 0.001). With the power of 100–175 mW and 20–40 ms’ duration, 35–128 laser shots were applied to the study eyes according to the number of targeted drusen.

**Table 1:** Demographics and clinical characteristics at baseline.

Characteristics	Value	*p*-value
No. of patients	20	
Gender (male:female)	2:18	
Age (year)	72.3 ± 5.6	
Study eye (OD:OS)	13:7	
**BCVA (letter)**		
Study eye	70.7 ± 8.9	< 0.001
Control eye	77.1 ± 7.0	
**Drusen area (mm**^**2**^**)**		
Study eye	3.4 ± 2.6	0.244
Control eye	3.1 ± 2.5	
**PED height (μm)**		
Study eye	146.5 ± 78.2	0.051
Control eye	111.3 ± 37.0	
**Drusen with pigmentation**		
Study eye	16/20 (80.0%)	1.000
Control eye	16/20 (80.0%)	
**Hyperreflective foci on SD-OCT**		
Study eye	5/20 (25.0%)	0.429
Control eye	3/20 (15.0%)	

Values are presented as the mean ± standard deviation or number.

*BCVA* best-corrected visual acuity, *PED* pigment epithelial detachment, *SD-OCT* spectral-domain optical coherence tomography.

### Primary outcomes

**Figure 2:**
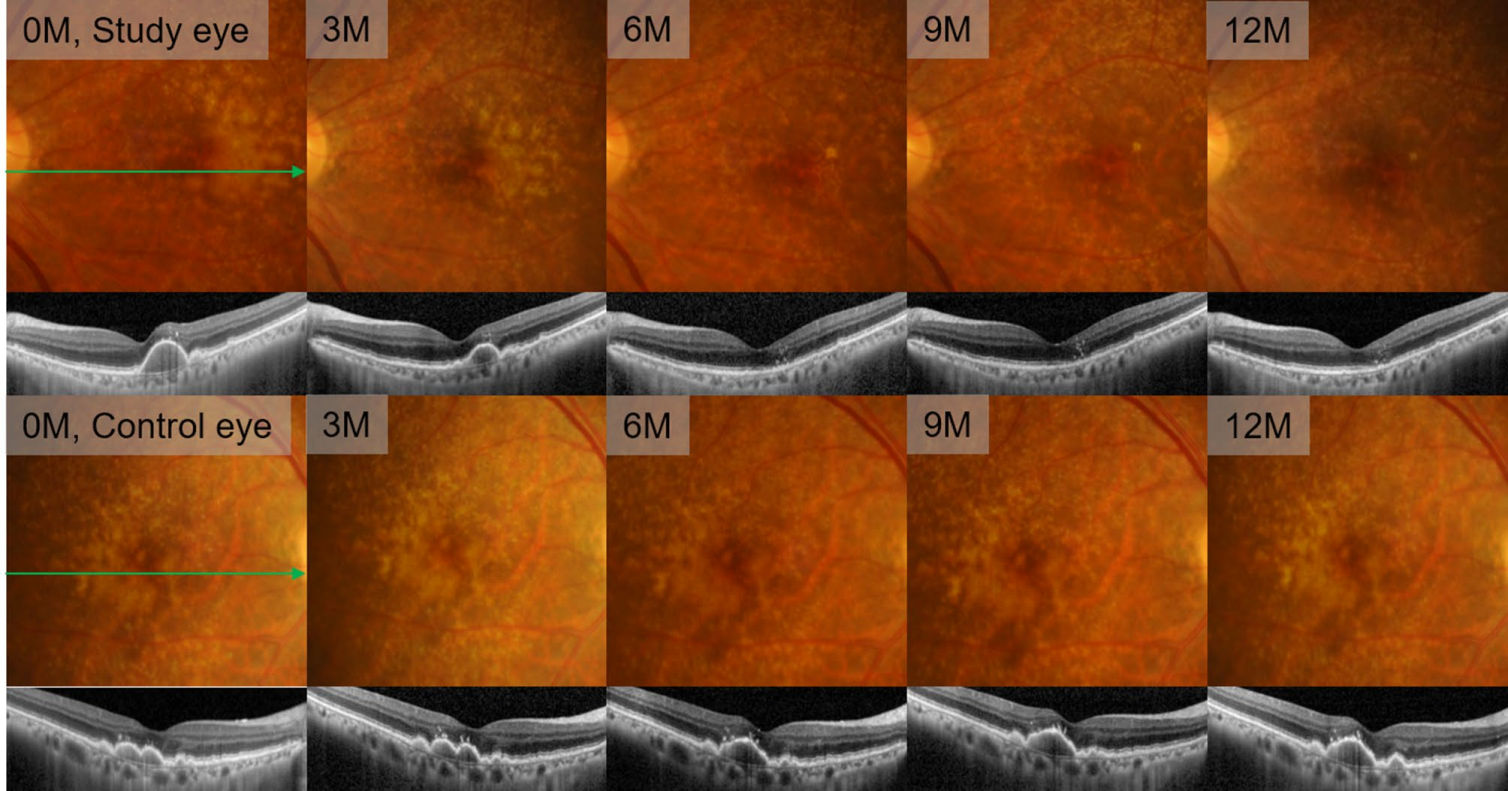
Serial color fundus photography and spectral-domain optical coherence tomography (SD-OCT) images from the left study eye (upper two rows) and right control eye (lower two rows) of a 69-year-old female patient (Case 7). Fundus photography shows gradual reduction of drusen area in study eye, while no change in control eye. 6 months after laser, SD-OCT images in study eye reveals complete collapse of pigment epithelial detachment (PED), while PED in control eye shows confluent appearance. PED did not recur during the 12 months follow-up in study eye.

**Figure 3:**
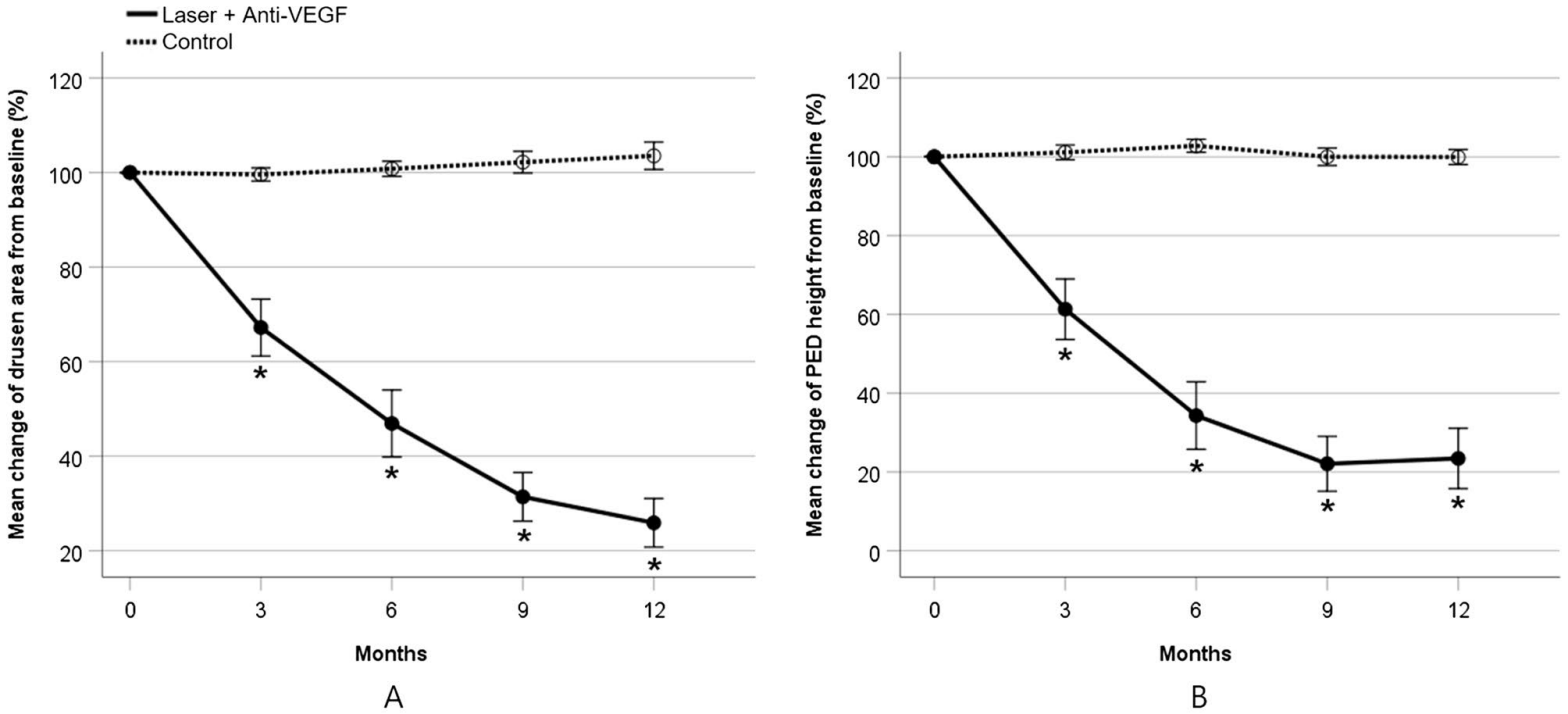
Mean change of (**A**) drusen area and (**B**) retinal pigment epithelial detachment (PED) height from baseline in study eye and control eye. VEGF = vascular endothelial growth factor, *P < 0.05.

The mean drusen area decreased in the study eye, while no difference showed in control eye (Fig. 2, Supplementary Fig. S2 online). The drusen area decreased by 74.1% in study eye (*P* < 0.001), while no significant change in control eye at 12 month examination (*P* = 0.210) (Fig. 3A, Table 2).

**Table 2:** Drusen area (mm^2^) in study eye and control eye during 12 months follow-up.

No	Study eye					Control eye				
	0 M	3 M	6 M	9 M	12 M	0 M	3 M	6 M	9 M	12 M
1	1.19	0.60	0.62	0.57	0.44	2.15	2.24	2.41	2.14	2.51
2	1.26	0.65	0.49	0.41	0.29	1.89	1.82	1.91	1.84	1.81
3	1.83	1.62	1.43	1.31	1.19	1.66	1.71	1.89	2.35	2.31
4	2.86	2.38	1.28	0.82	0.41	2.47	2.07	1.96	2.21	2.00
5	3.93	1.45	0.65	0.64	0.62	2.17	2.56	2.21	2.30	2.84
6	2.10	2.17	1.53	0.66	0.31	2.43	2.40	2.40	2.48	2.47
7	2.41	1.48	0.37	0.27	0.10	2.52	2.63	2.69	2.79	2.77
8	10.39	9.90	9.69	7.46	3.86	12.16	11.83	12.10	11.69	11.24
9	7.87	4.22	2.18	2.05	2.02	4.92	4.99	5.06	4.78	5.22
10	2.85	0.40	0.17	0.17	0.23	1.08	1.01	1.17	1.11	1.07
11	2.43	0.63	0.12	0.09	0.09	1.83	1.86	1.84	1.91	1.94
12	1.09	1.08	0.49	0.30	0.22	0.91	0.92	0.84	0.91	0.88
13	1.71	1.57	1.50	0.55	0.42	1.75	1.67	1.67	1.76	1.70
14	3.95	3.86	3.80	3.42	3.97	3.91	3.84	4.01	3.95	3.95
15	1.63	1.48	1.32	0.51	0.47	1.74	1.72	1.77	1.75	1.76
16	2.41	1.52	1.17	0.94	0.91	2.15	2.09	2.19	2.14	2.12
17	6.37	2.66	1.07	0.80	0.69	5.87	5.85	5.60	5.55	5.58
18	2.23	0.87	0.22	0.22	0.21	2.54	2.49	2.58	2.57	2.55
19	8.05	6.62	6.58	2.70	2.57	5.65	5.65	5.64	5.63	5.64
20	2.13	1.54	0.43	0.17	0.14	2.75	2.73	2.74	2.73	2.76

**Figure 4:**
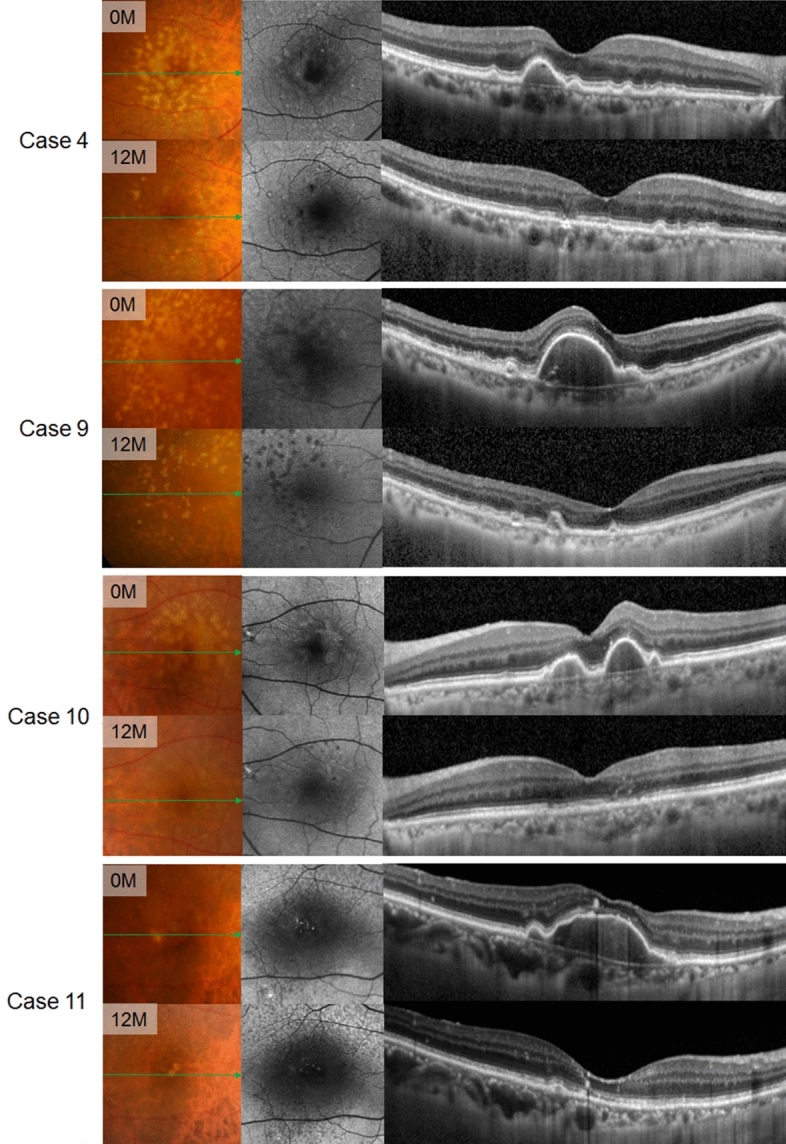
Serial color fundus photography (First column), fundus autofluorescence (Second column), and spectral-domain optical coherence tomography (Third column) scans in 4 representative eyes treated with laser. Dramatic resolution of pigment epithelial detachment were found over 12 months.

The mean PED height also decreased by 76.6% in study eye over 12 months (*P* < 0.001). In the control eye, however, PED height showed no reduction over 12 months (*P* = 0.971) (Fig. 3B, Supplementary Table S1 online). By 12 months, complete collapse of PED was observed in 9 of 20 (45%) study eyes (Fig. 4). In the serial SD-OCT B-scan images crossing through the points where the laser was applied, the hyperreflective dots derived from RPE layer were formed along with PED collapse. These hyperreflective dots gradually disappeared, and the RPE layer was restored (Supplementary Fig. S3 online).

### Secondary outcomes

**Figure 5:**
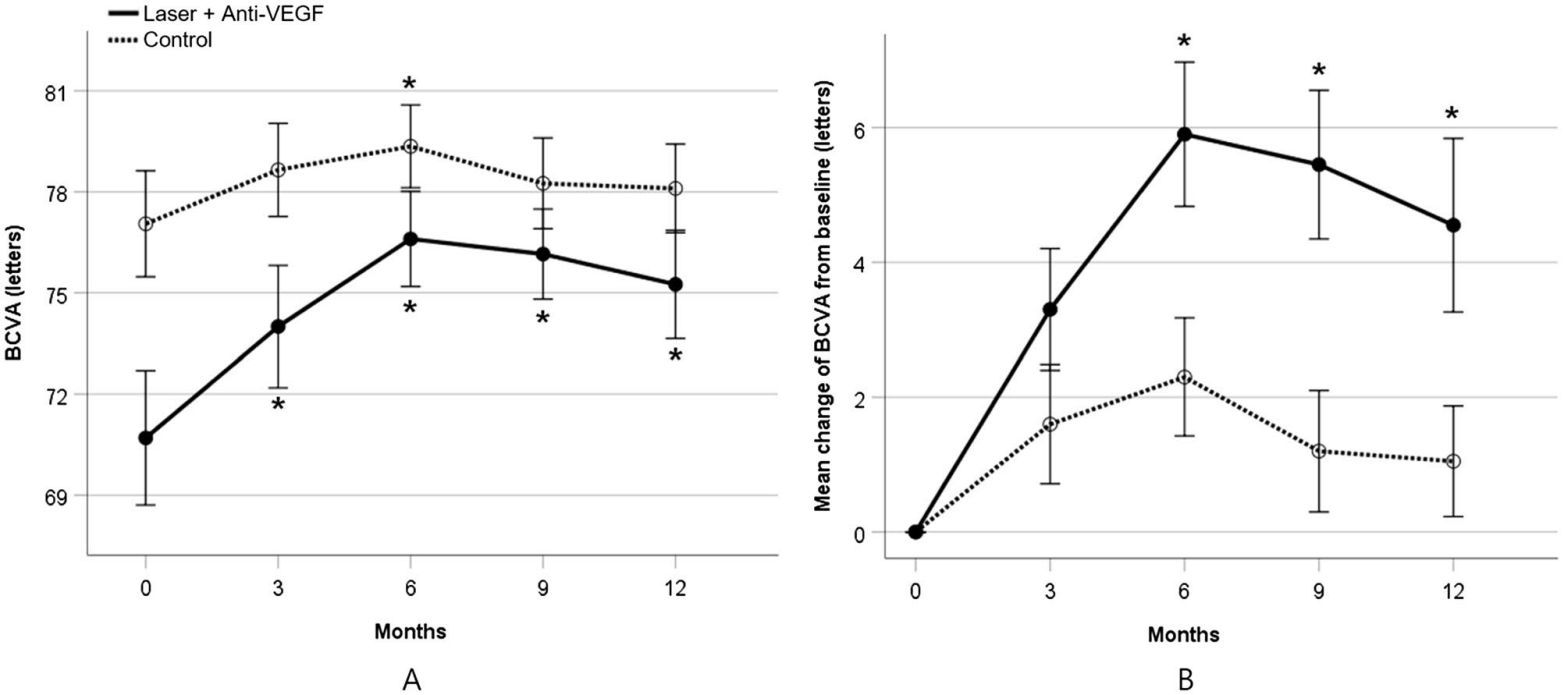
Comparison of (**A**) visual acuity and (**B**) visual gain between study eye and control eye in drusenoid pigment epithelial detachment. BCVA = best-corrected visual acuity, VEGF = vascular endothelial growth factor, *P < 0.05.

Compared with baseline, a significant BCVA improvement was seen in study eyes at month 3, 6, 9 and 12 (*P* < 0.001), while control eyes showed no significant difference except at month 6 (*P* = 0.063, 0.007, 0.172, 0.190, respectively) (Fig. 5A, Table 3).

**Table 3:** Best-corrected visual acuity (letters) in study eye and control eye during 12 months follow-up.

No	Study eye					Control eye				
	0 M	3 M	6 M	9 M	12 M	0 M	3 M	6 M	9 M	12 M
1	73	74	78	74	76	80	78	78	79	79
2	80	84	85	84	84	81	83	84	84	84
3	73	77	73	73	72	79	77	75	79	75
4	77	83	83	80	81	80	83	86	83	83
5	81	83	83	83	83	77	79	79	78	79
6	77	80	83	81	84	83	88	88	87	84
7	69	66	78	80	76	72	68	72	67	67
8	78	78	80	80	78	79	84	81	78	84
9	65	67	74	69	64	88	89	88	86	81
10	78	78	79	79	83	79	80	80	77	79
11	58	69	70	69	67	67	68	71	68	70
12	68	65	65	70	61	71	71	79	72	71
13	74	74	78	77	79	80	79	79	80	79
14	77	77	80	81	76	80	80	83	84	83
15	59	69	71	73	72	65	71	70	68	70
16	45	50	60	63	64	59	73	72	73	68
17	74	78	81	84	84	82	85	84	84	84
18	65	70	76	68	70	76	74	78	76	77
19	75	79	76	78	75	78	80	76	82	81
20	68	79	79	77	76	85	83	84	80	84

Mean change from baseline in BCVA was always higher in study eye compared with control eye, but was not significant at month 3 (*P* = 0.168). At 12 month examination, the change from baseline in BCVA was 4.6 letters in study eyes which was 3.5 letters better than those of the control eyes (*P* = 0.019) (Fig. 5B).

In both eyes, contrast sensitivity did not improve from baseline to month 3 and 12 in all spatial frequencies (Supplementary Table S2 online). The horizontal and vertical metamorphopsia scores in M-charts did not improve from baseline to all time points in both study and control eyes (Supplementary Table S3 online). NEI-VFQ 25 total score was 74.2 ± 17.4 at baseline, and showed no significant difference from that of month 12 (75.4 ± 16.5, *P* = 0.664).

Over the 12 months of follow up, 10 (50%) out of 20 study eyes showed nGA on SD-OCT and one case among them developed GA on color fundus photography, which did not affect the foveal center (Supplementary Fig. S4 online). There were no nGA development in control eyes during follow up.

On FAF, 14 (70%) out of 20 study eyes showed focal hyper-autofluorescence at laser sites and gradually faded or changed into hypo-autofluorescence (6 cases) over 12 months. Two eyes (10%) showed focal hypo-autofluorescence and 4 eyes (20%) showed no gross change over 12 months of follow up on FAF (Supplementary Fig. S5 online). Finally, 8 (40%) out of 20 study eyes showed hypo-autofluorescent spots at month 12.

### Safety

Among the study eye, one case of RPE tear occurred at month 12, and one case of retinal angiomatous proliferation occurred in control eye at month 6 (Supplementary Figs. S6, S7 online). Both cases received treatment with additional anti-VEGF injections and maintained without active exudation. Otherwise, no side effects related to laser or anti-VEGF injection including conjunctival hemorrhage, eye pain, vitreous floater, increased intraocular pressure, endophthalmitis, traumatic cataract, retinal detachment, retinal hemorrhage or macular burn were found.

## Discussion

In this study, we investigated the 12 months outcomes of laser treatment and anti-VEGF injections for intermediate AMD to evaluate the response of drusenoid PED. Laser treatment in combination with VEGF inhibitors resulted in visual acuity improvement, reduced drusen area and decreased PED height compared to the fellow control eyes which showed no change.

Drusenoid PED is usually distinguished from the other types of PED such as serous or vascularized PED by its better prognosis^13^. However, in studies investigating long-term prognosis of drusenoid PED in age-related eye disease study (AREDS) and AREDS2 participants, late AMD also occurred to a considerable degree by 5 years; central GA (19%, 27.4%, respectively) , CNV (23%, 28.4%, respectively)^10, 11^. In a retrospective study in 2004, 61 eyes with drusenoid PED with a mean follow-up of 4.6 years also progressed to persistent PED (38%), GA (49%), and CNV (13%)^12^. These progression rates were significantly higher than that of eyes containing large drusen and pigmentary change without drusenoid PED^11^. Long-lasting PED, whether persisted or collapsed later, was associated with RPE atrophy and visual loss^10,13^. It has been proposed that the long-term separation of the RPE from the underlying Bruch’s membrane/choriocapillaris complex leads to RPE atrophy and subsequent photoreceptor loss (Supplementary Fig. S8 online)^14^. Therefore, it is reasonable to postulate that early intervention to reattach RPE to Bruch’s membrane might preserve the remnant RPE function and overlying photoreceptors. Studies with other treatment options such as photodynamic therapy or intravitreal anti-VEGF injections for drusenoid PED showed limited effect, included small number of subjects, or had no controls^20–22^.

There have been several studies of laser treatment for drusen to slow progression, but study focusing on drusenoid PED has not been performed. In a British study, laser treatment for PED did not improve the visual prognosis at 18 months and the result continued for 4 years^23,24^. The authors, however, mentioned that heterogenous group of patients with vascular PED was mistakenly recruited into the study. In the current study, we included intermediate AMD with drusenoid PED without CNV and GA, and laser treatment and anti-VEGF injections yielded a rapid collapse of drusenoid PED. Eyes showing flattened PED also had more visual gain than control eyes by 12 months.

In previous studies applying laser to drusen, despite attempts of various laser types and protocols, the development of CNV remained as a huge hurdle against applying this treatment in general. Subthreshold retinal laser therapy was found to be effective for reducing drusen, but did not show benefits in reducing development of CNV or GA^25^. One of the trials even suspended new patient recruitment owing to concern about laser-induced CNV^26^. To reduce this serious complication, we modified two treatment settings. First, laser using 532 nm frequency-double (Nd:YAG) solid state laser at a very short pulse duration was applied to the peripheral boundary of drusenoid PED. Short duration (20–40 ms) was expected to inflict less collateral retinal damage. Nd:YAG laser, which is highly absorbed by hemoglobin and melanin in RPE, has also been found to be more safe for macular treatment than conventional laser type^27^. Second, we treated the laser eye with 3 monthly prophylactic intravitreal anti-VEGF injections to reduce the development of CNV. During 12 months follow-up, no development of CNV was reported except one case of RPE tear at month 12. The patient with RPE tear continued to treat with intravitreal anti-VEGF injections, and stabilized. Development of RPE tear was reported to account for 10% of natural history of PED^13^, which means that the mechanism of RPE tear in this case could be either laser-induced complication or a consequence of the natural history of the disease.

In this study, we found 10 (50%) cases of nGA among study eyes, and 1 (5%) case developed GA during the 12-month follow up. In a previous study, nGA was regarded as a prodromal lesion where drusen subsequently developed areas of atrophy on SD-OCT^19^. FAF characteristics of nGA showed both hyper- and hypo-autofluorescent changes in a previous study and our study, indicating that nGA is a lesion undergoing atrophic changes^28^. Therefore, it is necessary to follow up nGA lesions in this study whether nGA will develop into GA later on. At 12 months, small hypo-autofluorescent spots in FAF, which were suspected as laser-induced atrophy, were found in 8 study eyes (case 4, 9, 10, 13, 14, 15, 18, and 19, Supplementary Fig. S2 online). However, considering that there were far fewer hypo-autofluorescent spots in FAF compared to the total number of the laser applied—the control eye also has hypo-autofluorescent spots as drusen decreases (case 9, 10, 11, and 15)—this FAF finding is presumed to reflect not only RPE damage by laser but also a sequela of rapid PED resolution. Further research for the laser effect and damage is needed with long-term follow up. Nevertheless, because there was little clinical GA (only one case, Supplementary Fig. S4 online), and there was no nGA invading the foveal center, the significant damage caused by laser has not yet been manifested until 12 months follow up.

Previous studies evaluated drusen reduction by counting the number of eyes showing at least a 50% reduction of drusen area from the baseline. Despite some complementation, this protocol is not strictly objective because the judgment of drusen size relies entirely on the examiner. Therefore, we measured the drusen area and height of drusenoid PED with software to evaluate lesions quantitatively. In this way, detailed change of drusenoid PED size could be calculated. The drusen area decreased to 67.2% in month 3, 46.9% in month 6, 31.4% in month 9, and 25.9% in month 12. The PED height also decreased to 61.3% in month 3, 34.3% in month 6, 22.1% in month 9, and 23.4% in month 12. We could not directly compare these values with the previous results that suggesting the proportion of patients with drusen size reduction of more than 50%. Our results demonstrated most of drusenoid PED disappears within 6 months.

Unlike BCVA, the result of NEI-VFQ 25 did not improve as we expected, probably since the test was done with both eyes while the treatment effect was limited to only one treated eye with lower visual acuity. However, other functional vision tests such as contrast sensitivity and M-charts also showed no significant differences during 12 months follow-up.

The exact mechanism of drusenoid PED regression is unclear. However, in the magnified images of the laser site, damage to the RPE layer by laser and reduction of the PED fluid were simultaneously observed at a relatively early phase. In all study eyes except for the complication cases, the recovery of the RPE layer occurred at the same time as the PED reduced (Supplementary Fig. S3 online). One possibility is to speculate that after the activation and proliferation of RPE cells or macrophage via laser, enhanced phagocytosis occurs and the drusenoid PED components get absorbed. Further laboratory study is necessary to elucidate how laser treatment removes drusenoid PED material.

There are some limitations in this study. First, unlike our intention to observe the effects of laser to drusen, the anti-VEGF injections used to suppress CNV development might affect the course of drusenoid PED. However, drusenoid PED is thought to show a limited response to anti-VEGF therapy as we normally encounter in clinical practice^29^. Thus, the effect of anti-VEGF on drusenoid PED is expected to be minimal in this study. Second, although being a prospective study, this was conducted short-term in a single center more to serve as a pilot study for multi-center randomized trials. In AREDS2 subgroup study, time from drusenoid PED detection to appearance of central GA or neovascular AMD took 2.6 years and 2.0 years respectively^10^. This is a 1-year follow up study to elucidate effect of laser for drusenoid PED. Long term extension studies up to 5 years is scheduled after the 1 and 2-year study period. Third, if the fundus status (i.e. degree of drusenoid PED) were similar, eyes with lower VA were assigned to laser treatment. Thus, interpretation of the direct comparison of VA between treat and control eyes should be cautious because of the ceiling effect of eye with better vision.

In summary, this report is aimed to investigate the efficacy and safety of the laser and anti-VEGF treatment for drusenoid PED. In this study, the application of laser photocoagulation using low energy and anti-VEGF injections improve visual acuity and facilitate drusenoid PED regression without serious complication like center involving GA. Accompanied regular anti-VEGF injections might protect eyes from CNV. Based on these results, we concluded that this study could be continued for long-term follow up. The 2 year results of this study will be followed, and it may be necessary to observe patients more than 5 years, especially for the possible progression of nGA lesions among study eyes. Larger, randomized clinical trials with long term follow-up period should be followed to confirm the effect of this treatment protocol for intermediate AMD.

## Supplementary information

Supplementary Information 1.

## Data Availability

All data generated or analyzed during this study are included in this published article (and its Supplementary Information files).
