# Supplementary material for: Laser and anti-vascular endothelial growth factor treatment for drusenoid pigment epithelial detachment in age-related macular degeneration
Source: Sci Rep. 2020 Sep 1;10:14370. doi: 10.1038/s41598-020-71401-3 (PMC7462975; doi:10.1038/s41598-020-71401-3)
Supplement: Supplementary file 1 — Supplementary Information 1. [file 41598_2020_71401_MOESM1_ESM.docx]

**Supplementary Information**

**Laser and Anti-Vascular Endothelial Growth Factor Treatment for Drusenoid Pigment Epithelial Detachment in Age-Related Macular Degeneration**

Min Seok Kim, MD^1,2 *^; Na-Kyung Ryoo, MD^3 *^; Kyu Hyung Park, MD, PhD ^1,2 †^

^1^Department of Ophthalmology, Seoul National University College of Medicine, Seoul, Korea

^2^Department of Ophthalmology, Seoul National University Bundang Hospital, Seongnam, Korea

^3^Department of Ophthalmology, Veterans Health Service Medical Center, Seoul, Korea

^*^ Both authors (Kim MS, Ryoo NK) contributed equally to this work.

^†^ **Correspondence:**

Kyu Hyung Park, M.D., Ph.D.

Department of Ophthalmology, Seoul National University Bundang Hospital, 173-82 Gumi-ro, Bundang-gu, Seongnam-si, Gyeonggi-do, 13620, Korea

Tel: +82-31-787-7373, Fax: +82-31-787-4057, E-mail: jiani4@snu.ac.kr

**Supplementary Table S1. Pigment epithelium detachment height (μm)** **in study eye and control eye during 12-months follow-up**

|  | Study eye | | | | | Control eye | | | | |
| --- | --- | --- | --- | --- | --- | --- | --- | --- | --- | --- |
| No | 0 M | 3 M | 6 M | 9 M | 12 M | 0 M | 3 M | 6 M | 9 M | 12 M |
| 1 | 104 | 28 | 26 | 13 | 10 | 108 | 110 | 125 | 132 | 125 |
| 2 | 65 | 54 | 42 | 0 | 0 | 119 | 110 | 123 | 133 | 127 |
| 3 | 80 | 39 | 34 | 27 | 26 | 72 | 75 | 75 | 80 | 75 |
| 4 | 149 | 68 | 0 | 0 | 0 | 106 | 119 | 117 | 114 | 122 |
| 5 | 160 | 61 | 23 | 27 | 32 | 118 | 119 | 130 | 143 | 141 |
| 6 | 69 | 73 | 45 | 0 | 0 | 96 | 98 | 103 | 96 | 106 |
| 7 | 269 | 154 | 0 | 0 | 0 | 143 | 162 | 209 | 207 | 228 |
| 8 | 108 | 103 | 103 | 76 | 49 | 98 | 80 | 75 | 85 | 88 |
| 9 | 319 | 91 | 97 | 81 | 73 | 91 | 90 | 77 | 77 | 22 |
| 10 | 194 | 0 | 0 | 0 | 0 | 70 | 64 | 79 | 79 | 65 |
| 11 | 291 | 49 | 0 | 0 | 0 | 181 | 201 | 200 | 232 | 203 |
| 12 | 76 | 52 | 0 | 0 | 0 | 55 | 62 | 62 | 62 | 62 |
| 13 | 82 | 69 | 0 | 0 | 27 | 96 | 83 | 93 | 93 | 96 |
| 14 | 134 | 134 | 137 | 84 | 157 | 123 | 127 | 127 | 120 | 120 |
| 15 | 95 | 100 | 85 | 72 | 62 | 88 | 94 | 94 | 100 | 101 |
| 16 | 118 | 0 | 0 | 0 | 0 | 210 | 221 | 218 | 228 | 220 |
| 17 | 249 | 201 | 76 | 53 | 31 | 103 | 98 | 112 | 119 | 88 |
| 18 | 163 | 133 | 0 | 0 | 0 | 150 | 156 | 159 | 160 | 164 |
| 19 | 115 | 112 | 117 | 118 | 114 | 112 | 110 | 115 | 107 | 109 |
| 20 | 90 | 56 | 22 | 18 | 10 | 87 | 67 | 72 | 58 | 62 |

**Supplementary Table S2. Test results of contrast sensitivity in study eye and control eye**

| **Measurement** | **Contrast Sensitivity at Various Cycles per Degree** | | | | |
| --- | --- | --- | --- | --- | --- |
|  | **1.5** | **3** | **6** | **12** | **18** |
| **Study eye (n=20)** |  |  |  |  |  |
| Baseline | 33.6±18.5 | 40.0±29.2 | 16.4±21.1 | 3.6±7.7 | 0.8±1.9 |
| Month 3 | 30.8±13.4  (0.447) | 30.6±26.6  (0.184) | 18.4±19.4  (0.643) | 2.9±4.8  (0.705) | 0.9±2.5  (0.830) |
| Month 12 | 39.6±22.5  (0.283) | 39.7±29.5  (0.961) | 23.6±29.7  (0.298) | 5.1±6.0  (0.433) | 1.7±2.7  (0.173) |
| **Control eye (n=20)** |  |  |  |  |  |
| Baseline | 31.9±11.9 | 48.9±28.4 | 23.1±17.9 | 4.1±5.0 | 0.8±1.9 |
| Month 3 | 36.1±16.4  (0.160) | 40.1±23.3  (0.234) | 25.5±29.5  (0.596) | 3.6±4.3  (0.597) | 1.5±2.5  (0.114) |
| Month 12 | 34.6±17.1  (0.497) | 39.4±29.8  (0.147) | 25.1±22.7  (0.747) | 6.1±7.8  (0.261) | 2.1±2.7  (0.106) |

Data are presented as mean ± standard deviation. (p-value compared to baseline)

**Supplementary Table S3. Test results of M-chart in study eye and control eye**

|  | **Months** | | | | |
| --- | --- | --- | --- | --- | --- |
|  | **0** | **3** | **6** | **9** | **12** |
| **Study eye (n=20)** |  |  |  |  |  |
| Horizontal | 0.19±0.08 | 0.19±0.06  (1.000) | 0.34±0.10  (0.017) | 0.28±0.08  (0.270) | 0.20±0.04  (0.901) |
| Vertical | 0.23±0.09 | 0.25±0.09  (0.587) | 0.21±0.05  (0.769) | 0.20±0.07  (0.645) | 0.21±0.05  (0.809) |
| **Control eye (n=20)** |  |  |  |  |  |
| Horizontal | 0.19±0.07 | 0.11±0.05  (0.223) | 0.14±0.05  (0.433) | 0.15±0.05  (0.550) | 0.12±0.05  (0.276) |
| Vertical | 0.10±0.03 | 0.11±0.04  (0.813) | 0.12±0.04  (0.525) | 0.16±0.05  (0.095) | 0.16±0.04  (0.023) |

Data are presented as mean ± standard deviation. (p-value compared to baseline)


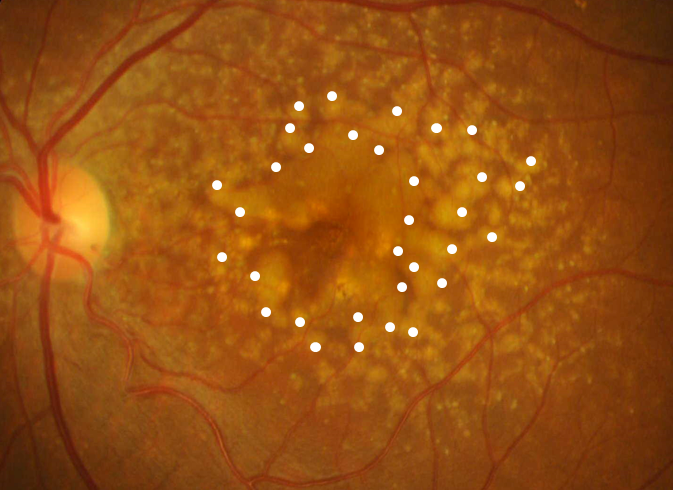


Supplementary Figure S1. Example of laser sites in eyes with drusenoid pigmented epithelial detachment. 35-128 laser shots of 20-40 milliseconds’ duration and 100 μm spot size at the lowest intensity to produce a nearly in-visible burn were applied along the periphery of drusenoid pigmented epithelial detachment.


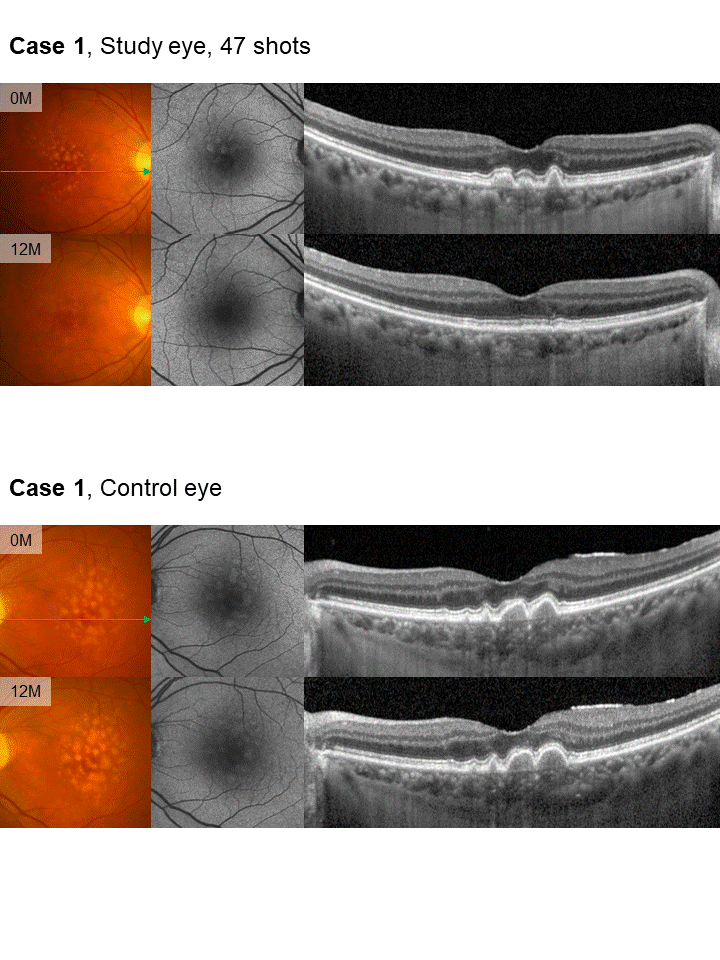


**
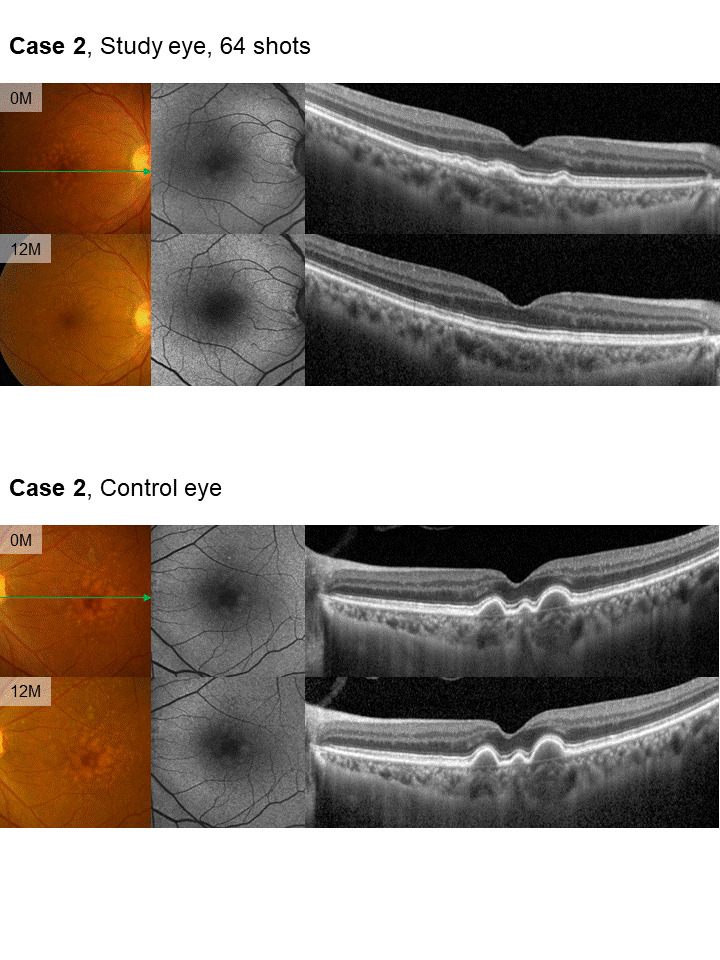
**

**
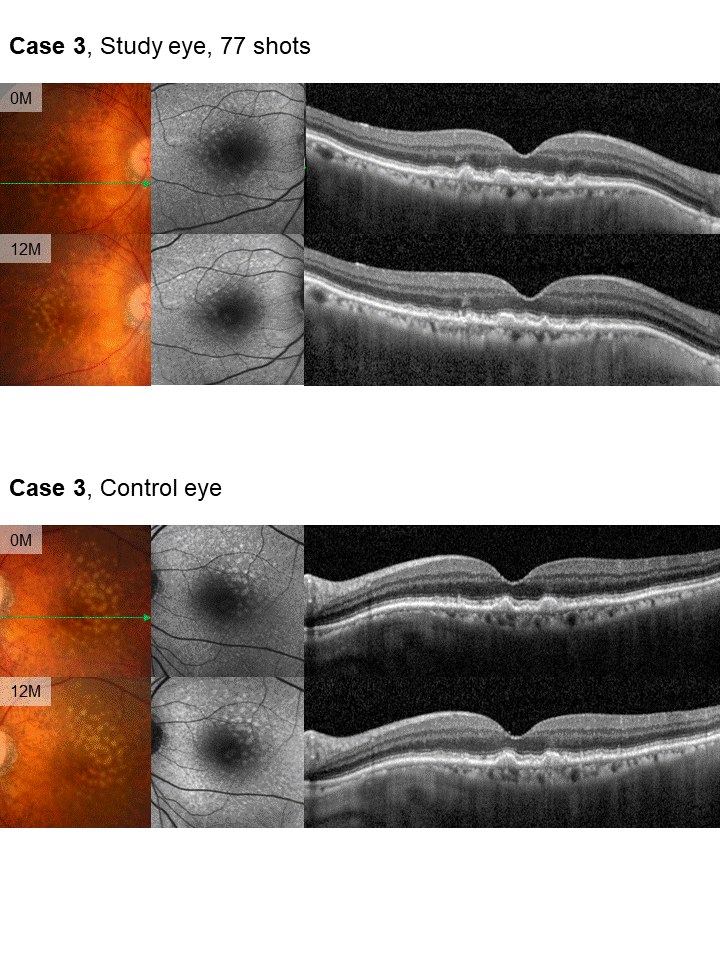
**

**
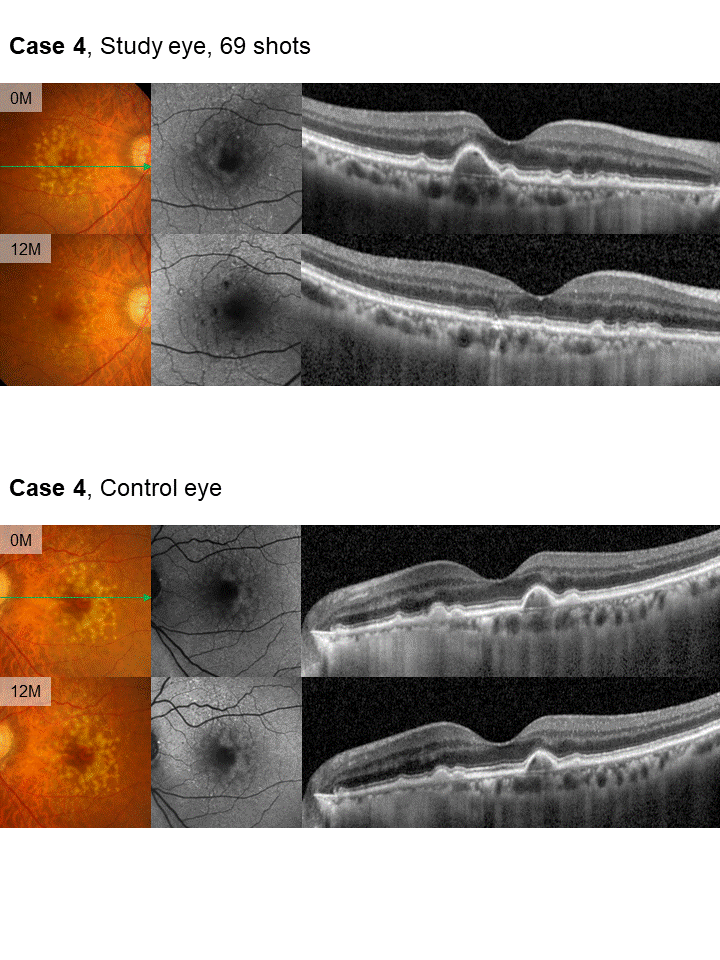
**

**
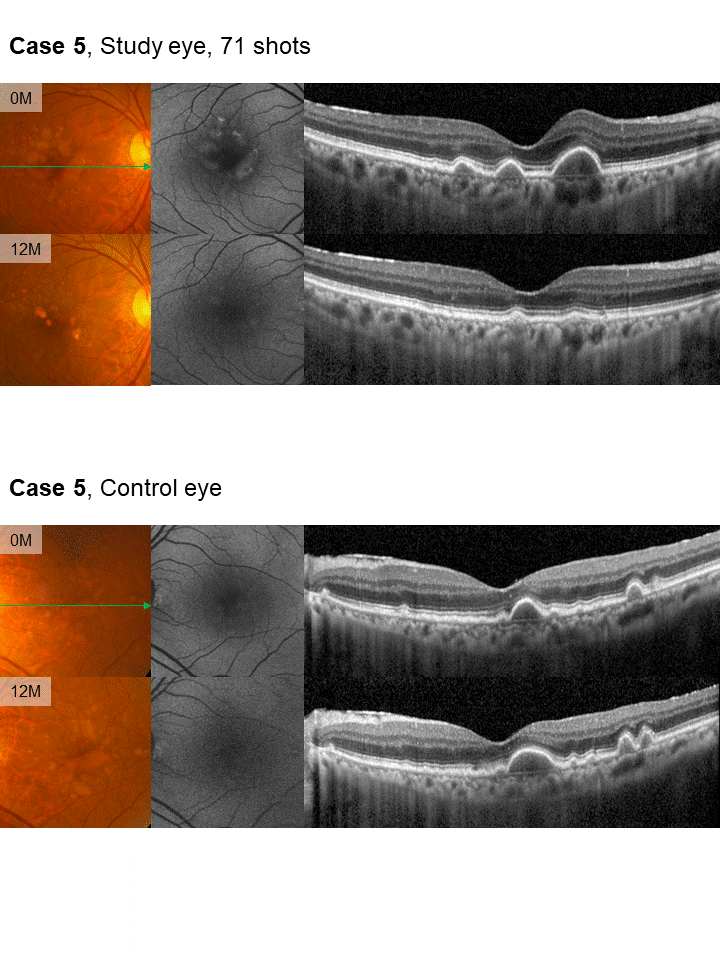
**

**
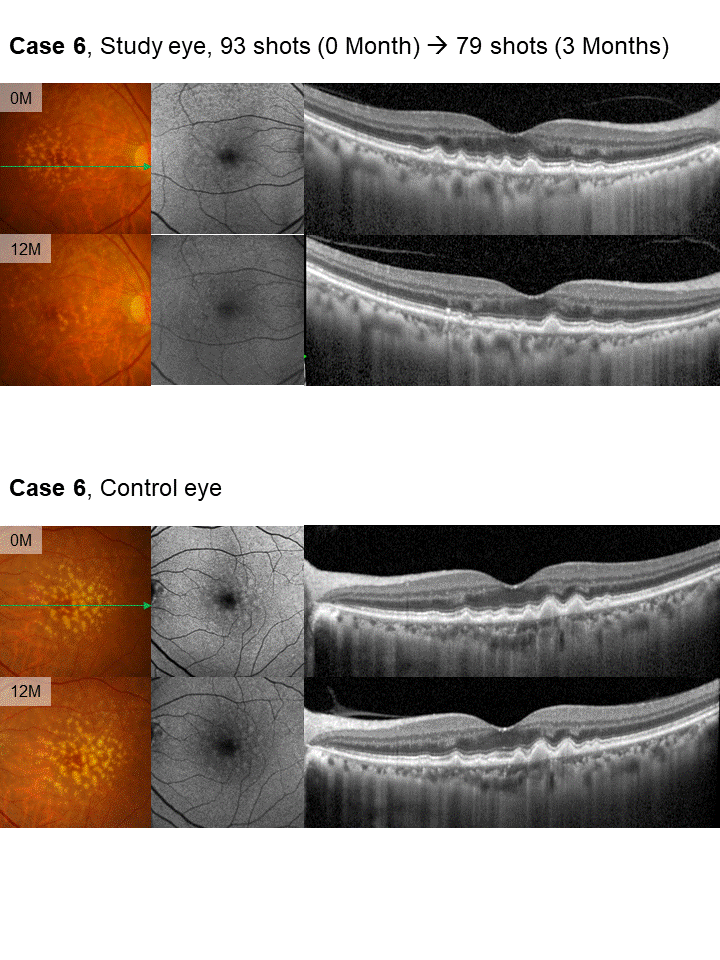
**

**
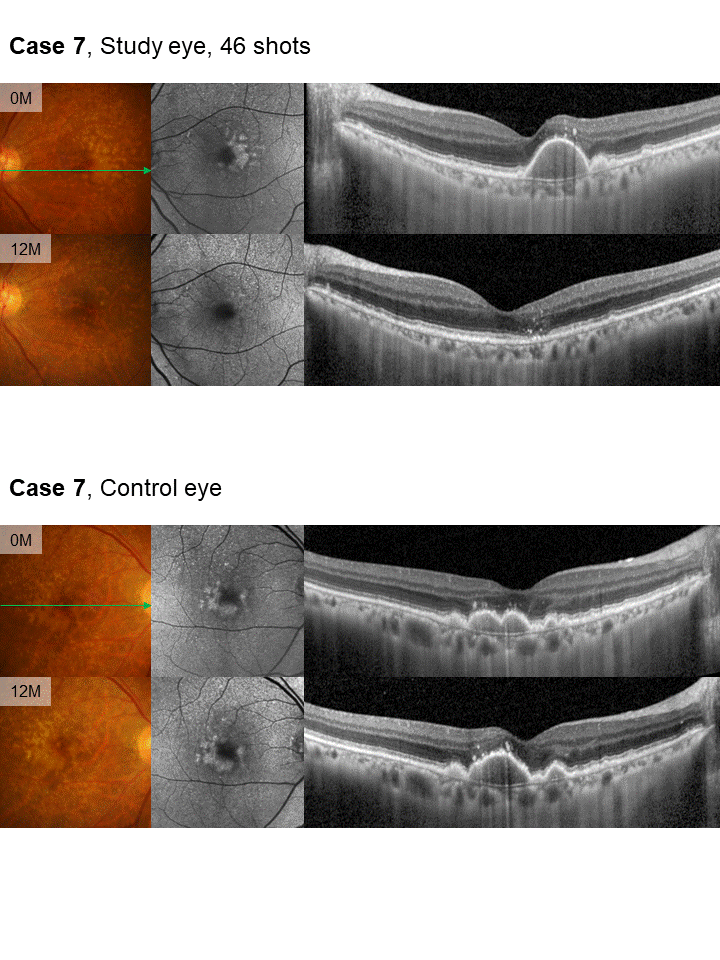
**

**
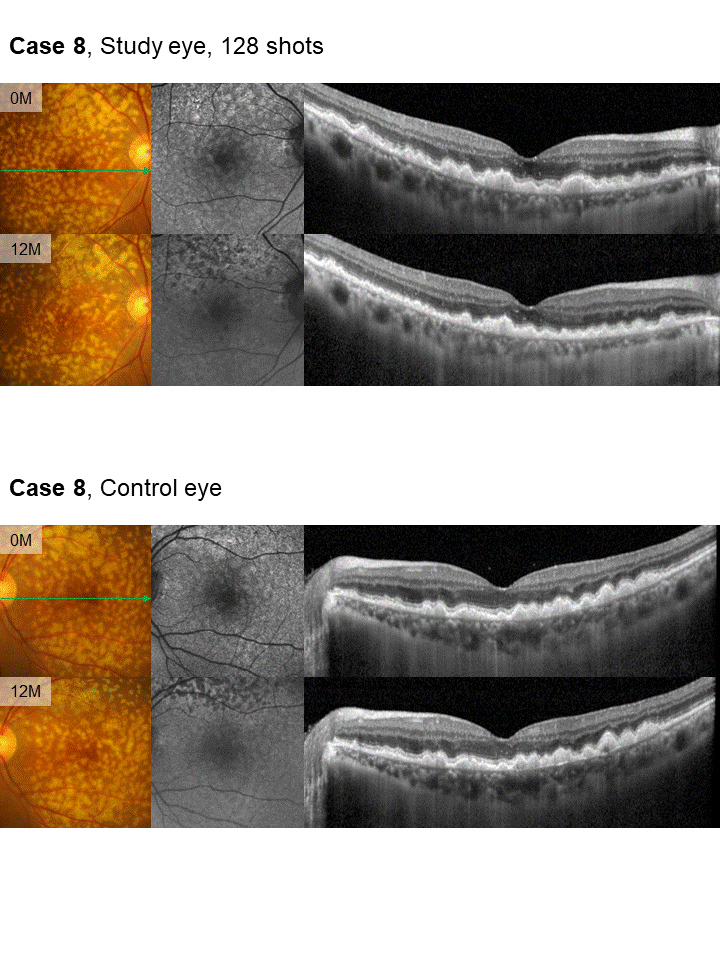
**

**
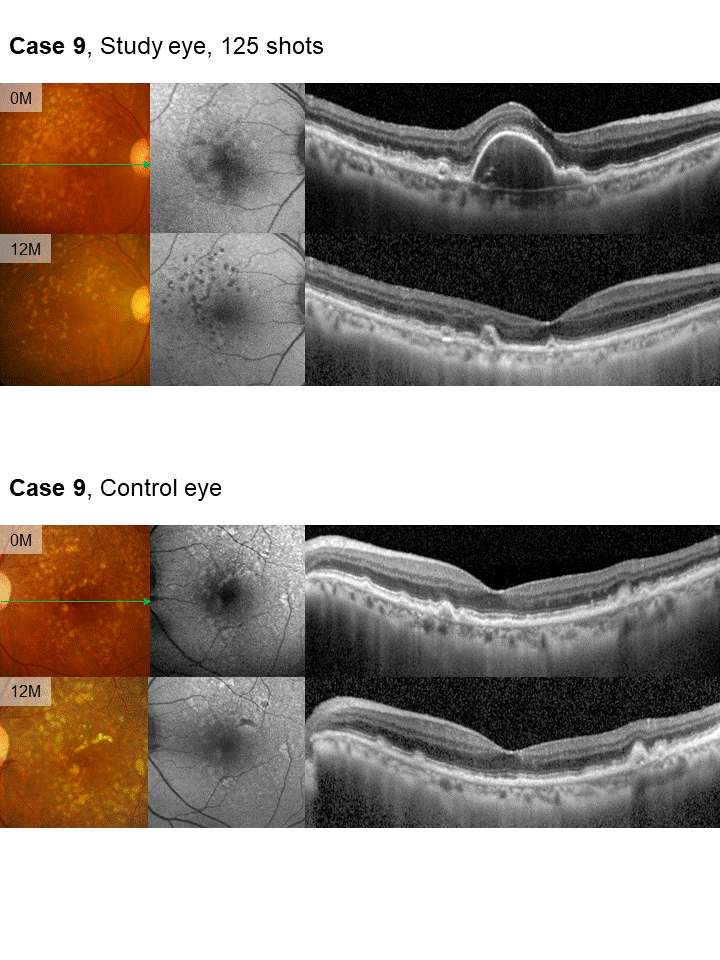

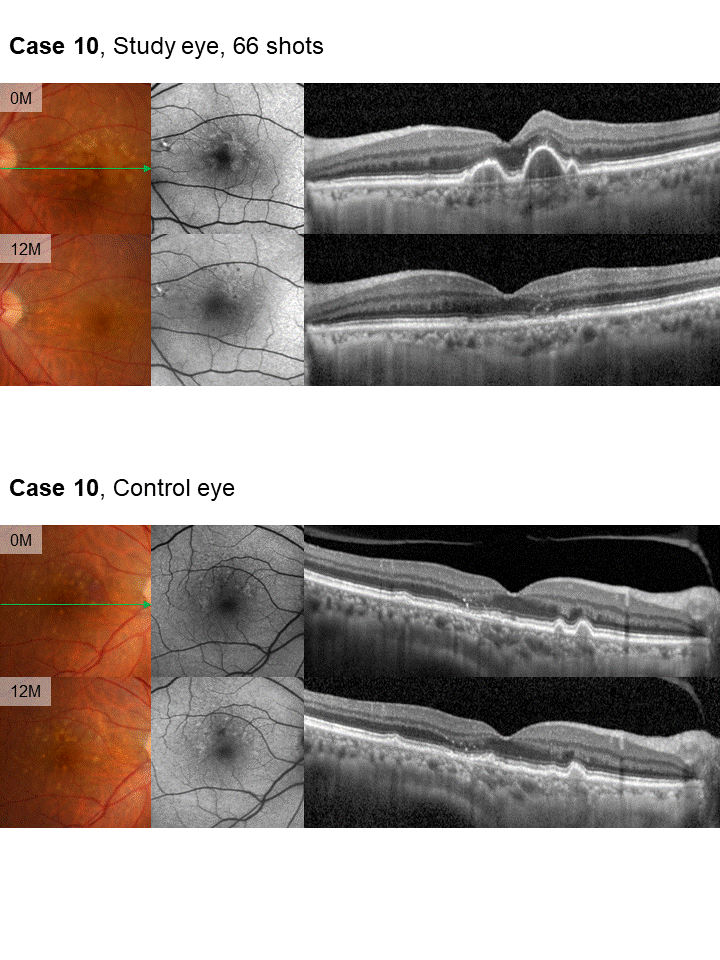
**

**
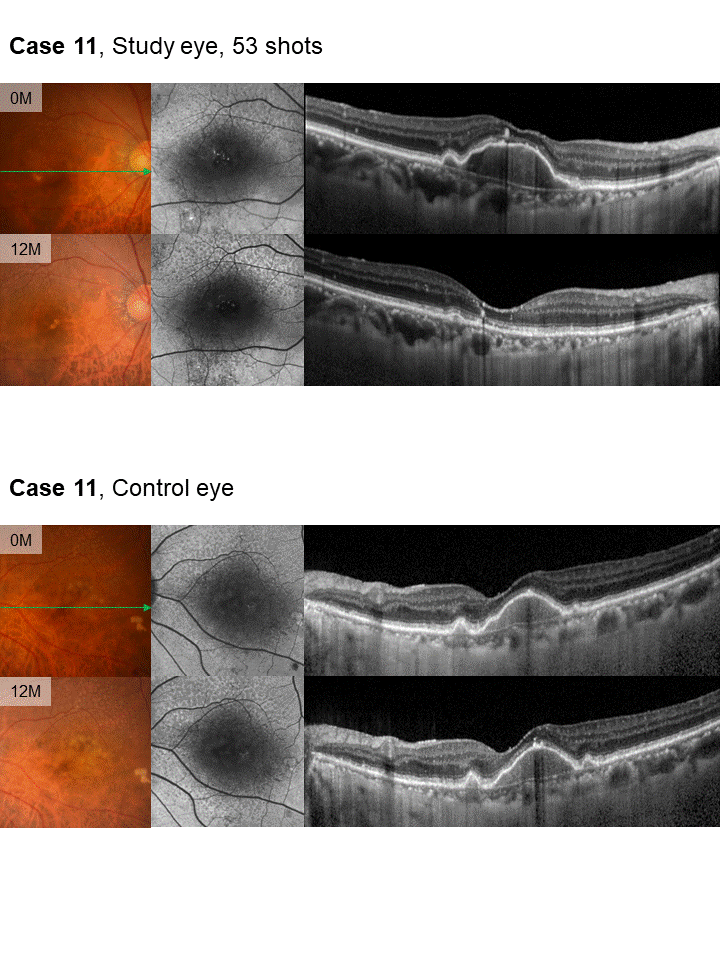

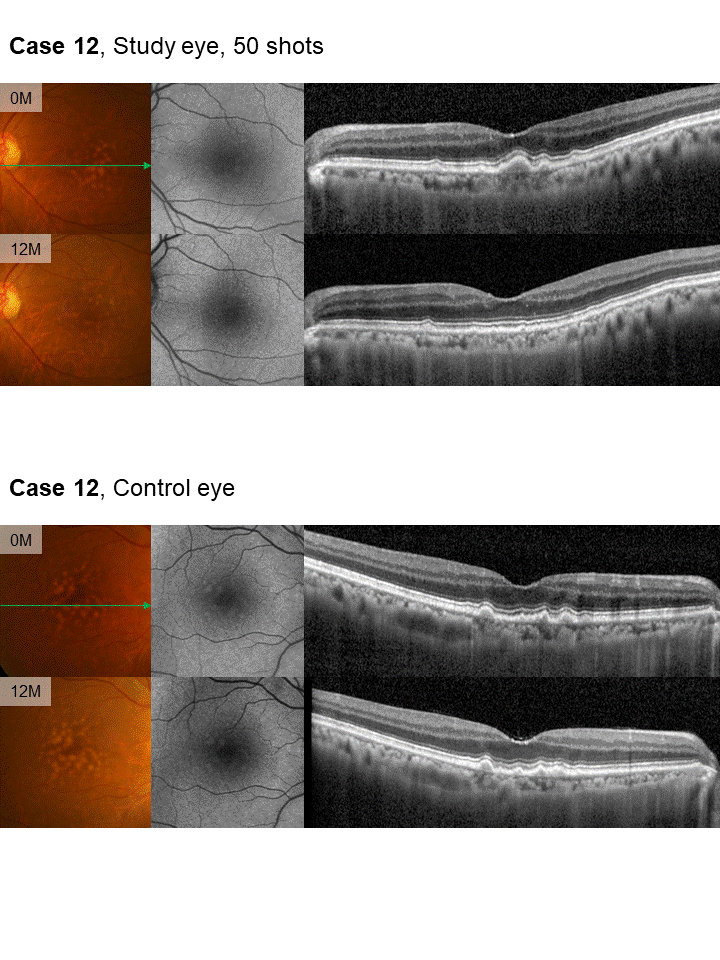

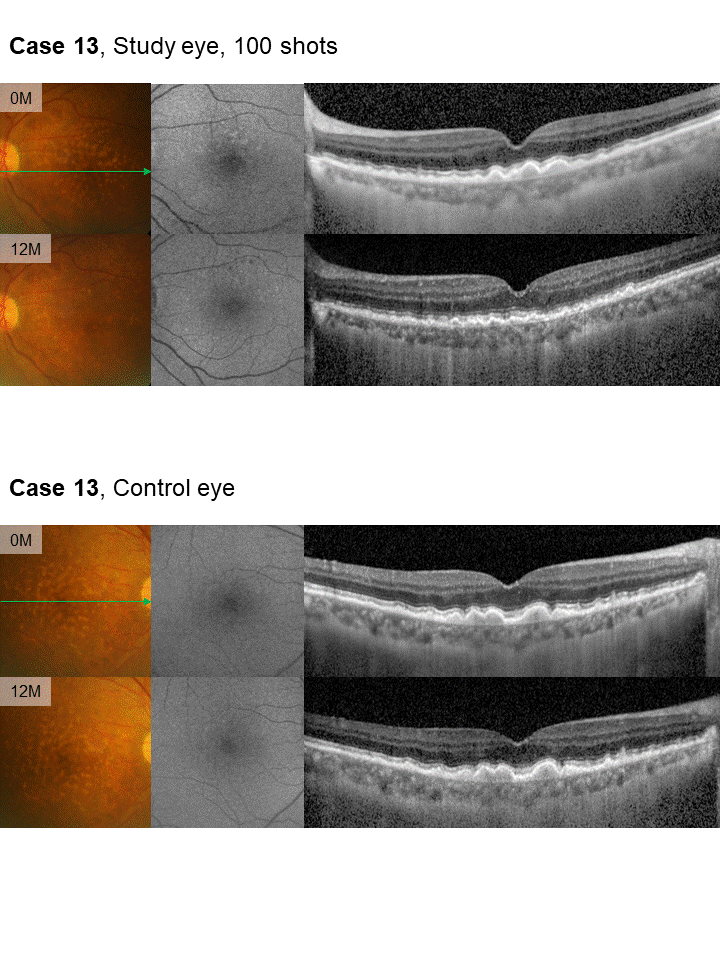
**

**
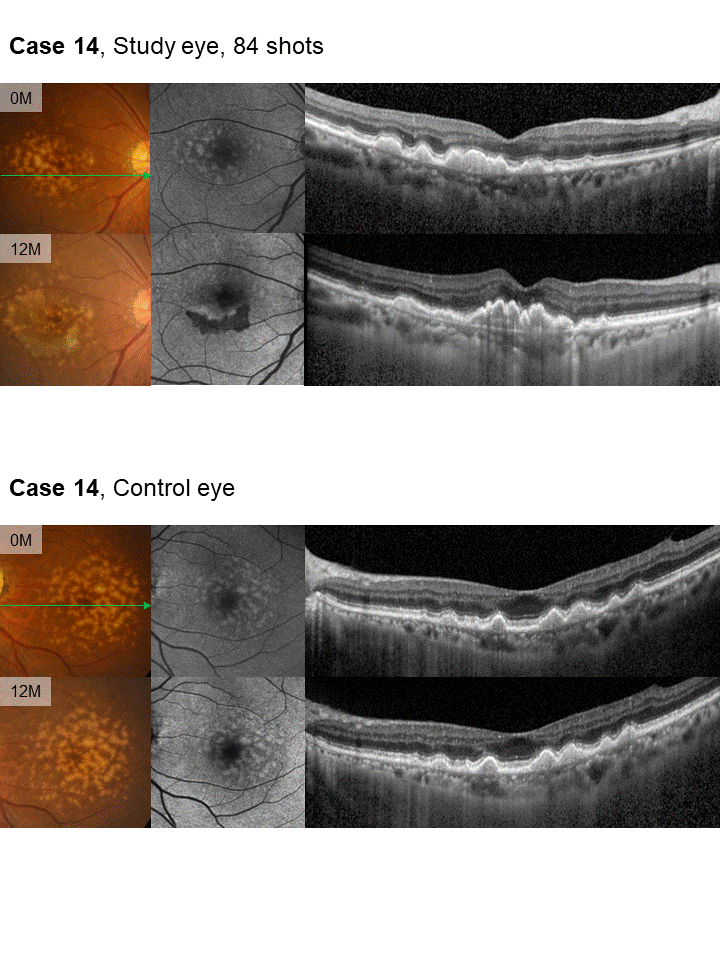

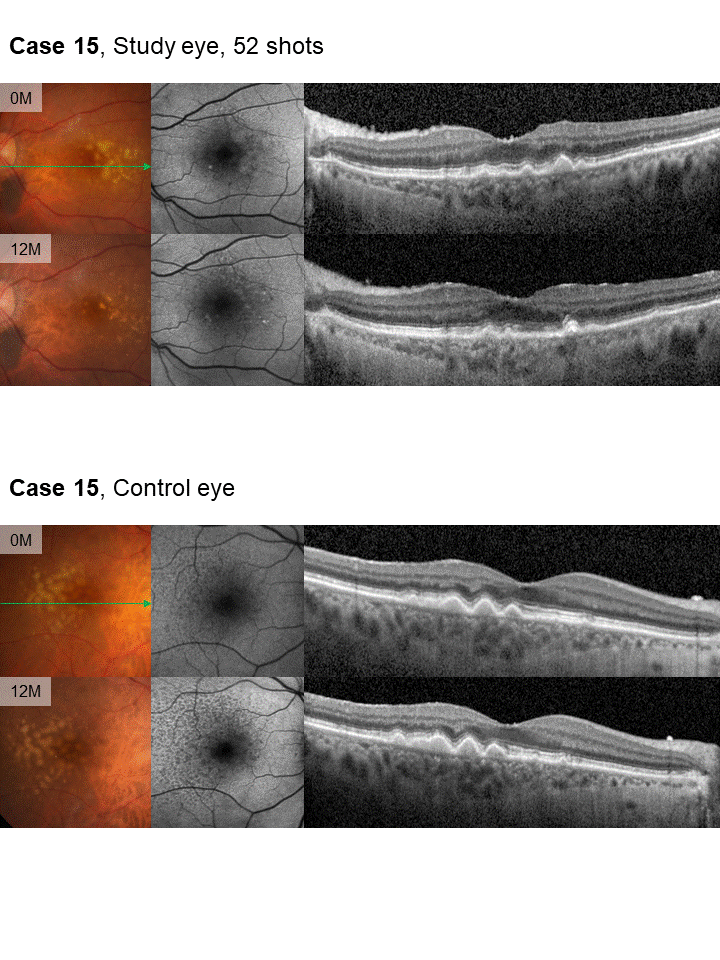

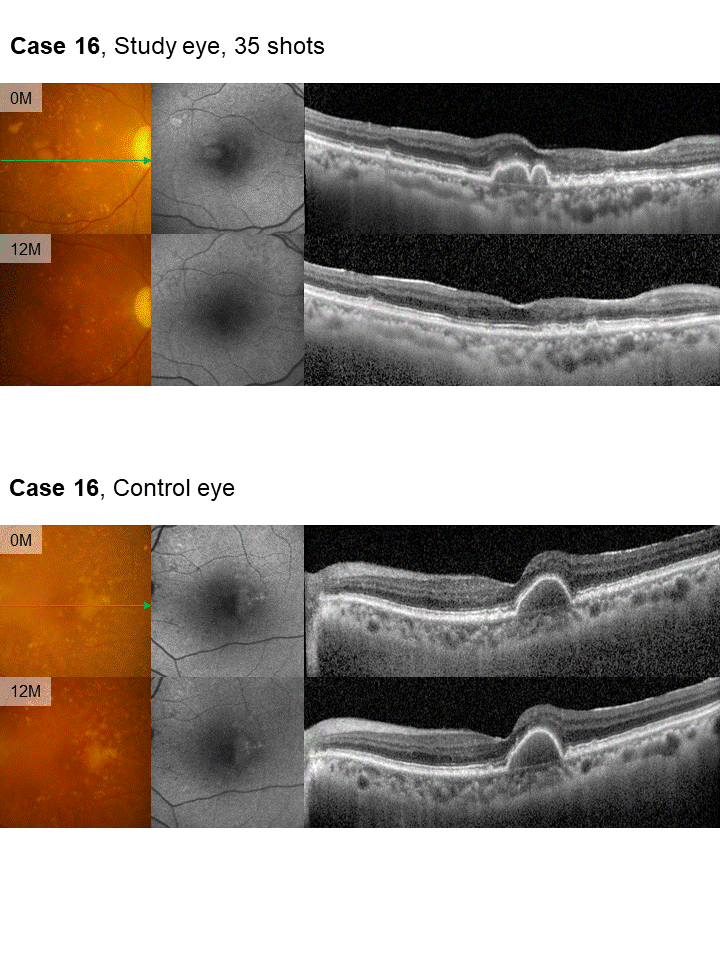

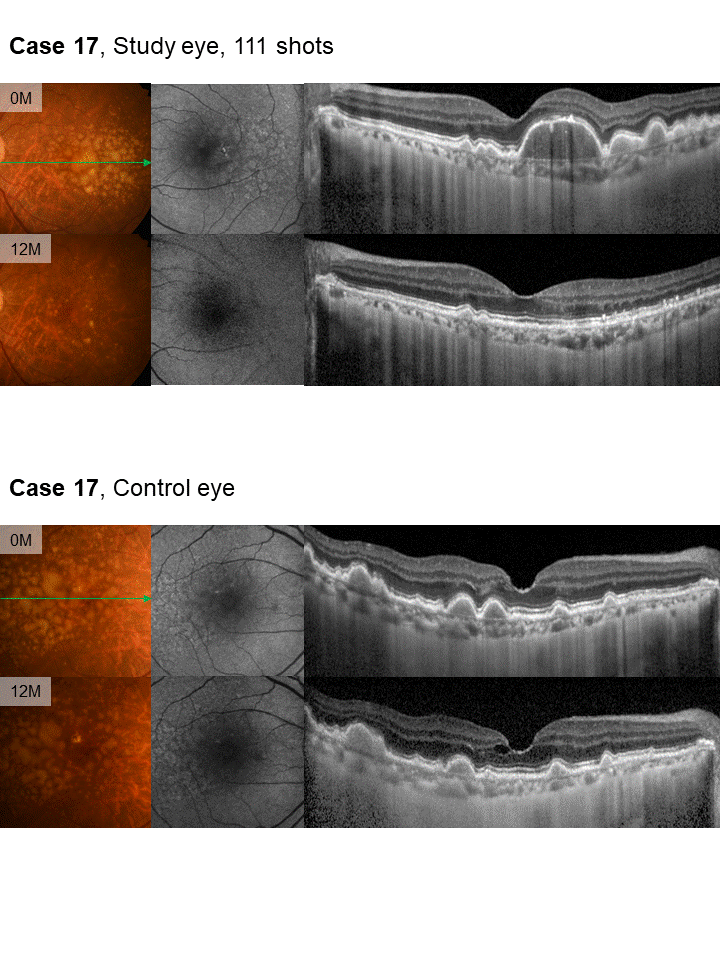
**

**
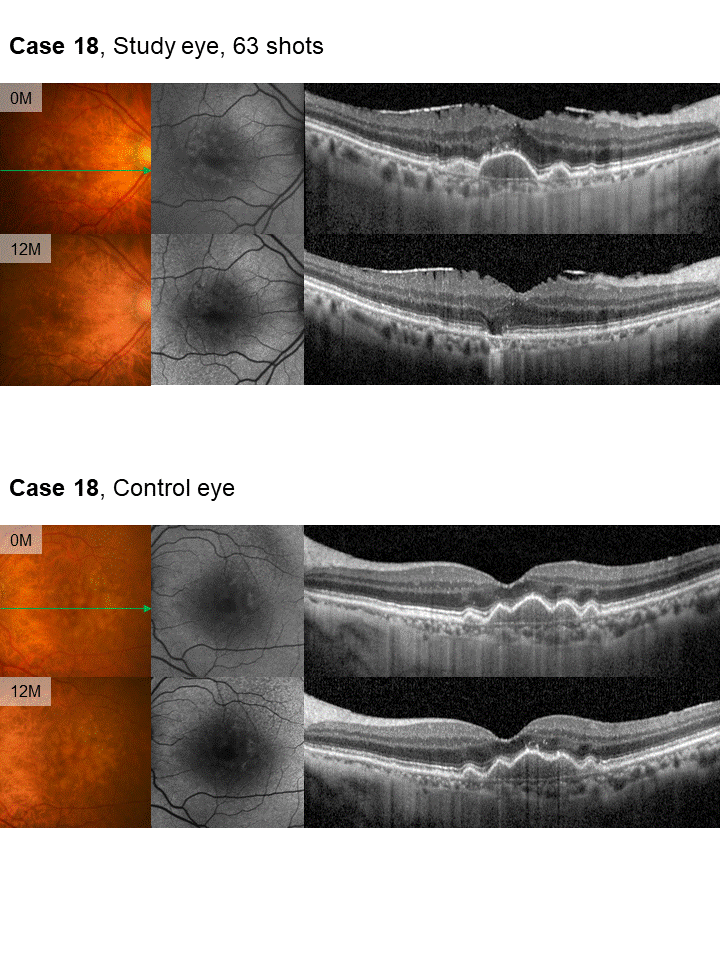

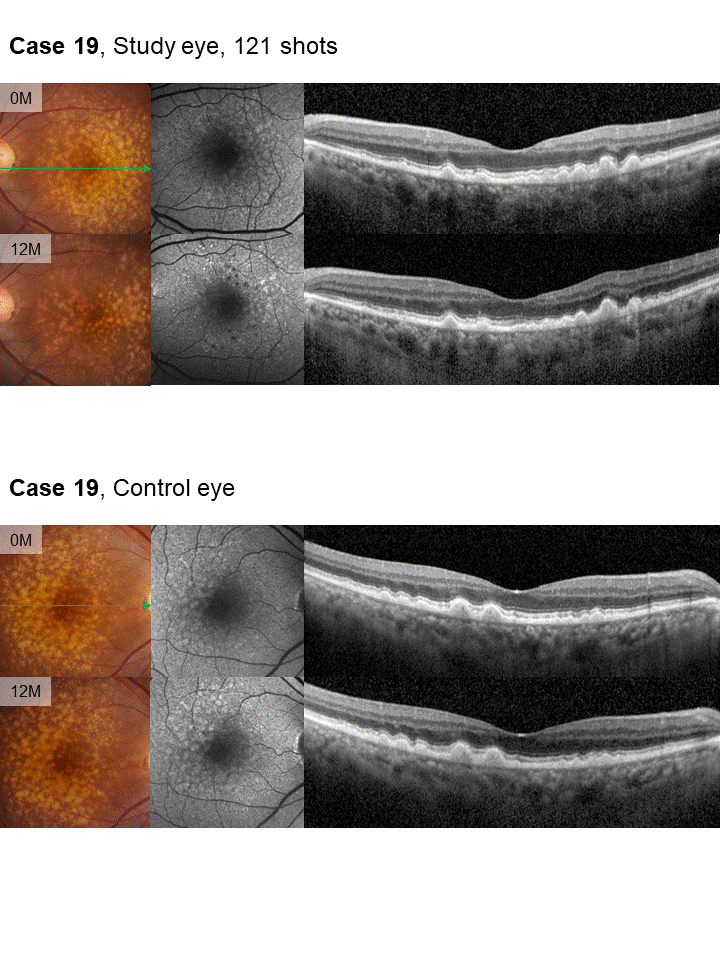
**

**
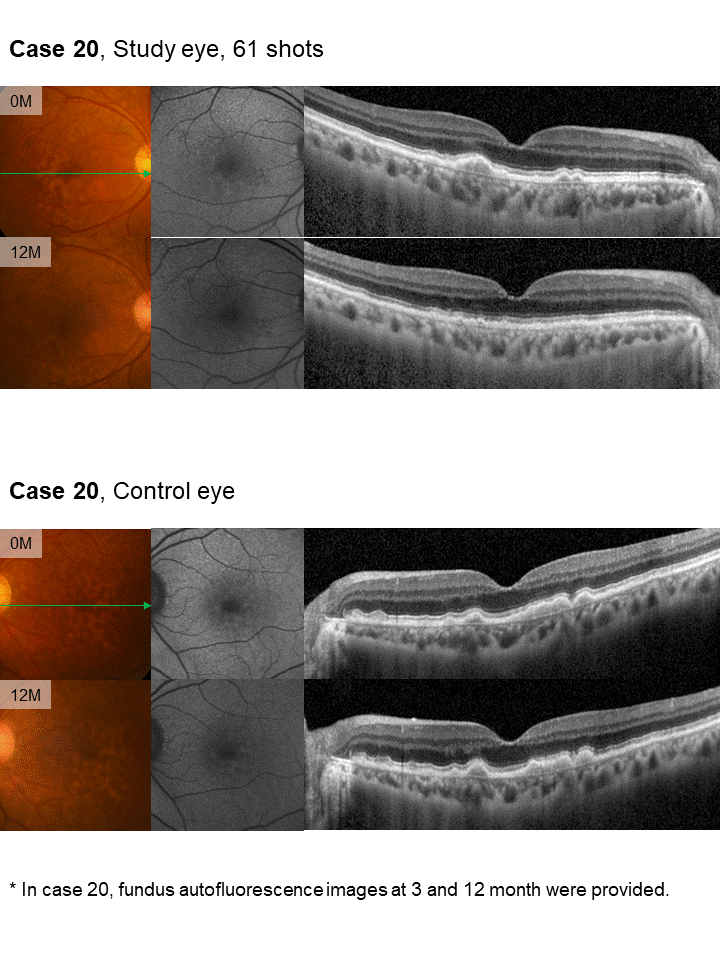
**

**Supplementary Figure S2.** Fundus photography, fundus autofluorescence, and optical cohorence tomography images of the all 20 study eyes and control eyes at the initial examination and at 12 months.


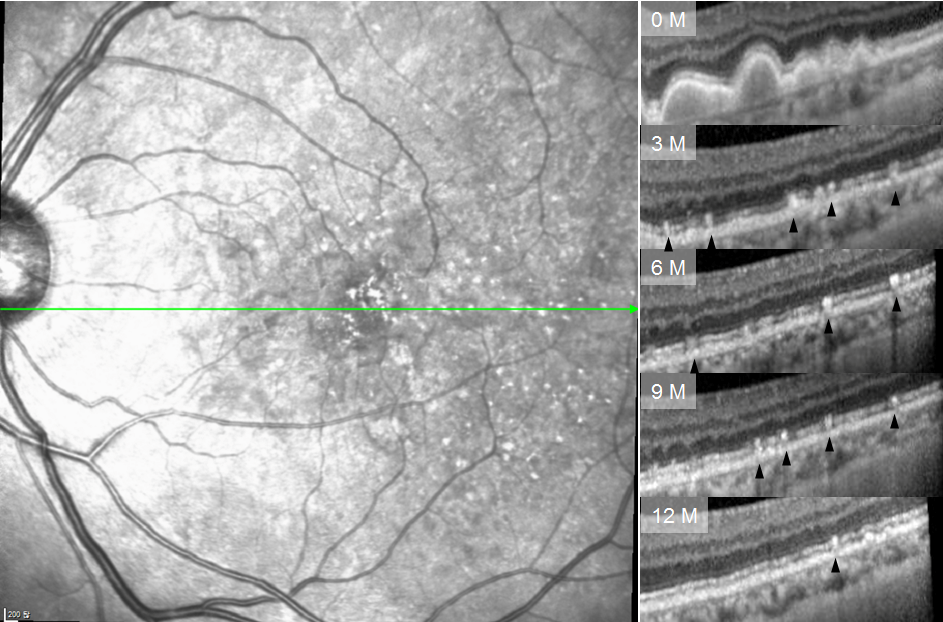


Supplementary Figure S3. Serial spectral-domain optical coherence tomography images crossing the laser points in the left eye of case 17. At month 3, retinal pigment epithelial detachment was totally collapsed, and laser sites appeared as hyperreflective dots arose from the retinal pigment epithelium (RPE) layer (black arrowhead). These hyperreflective dots were gradually disappeared along with the restoration of the RPE layer.


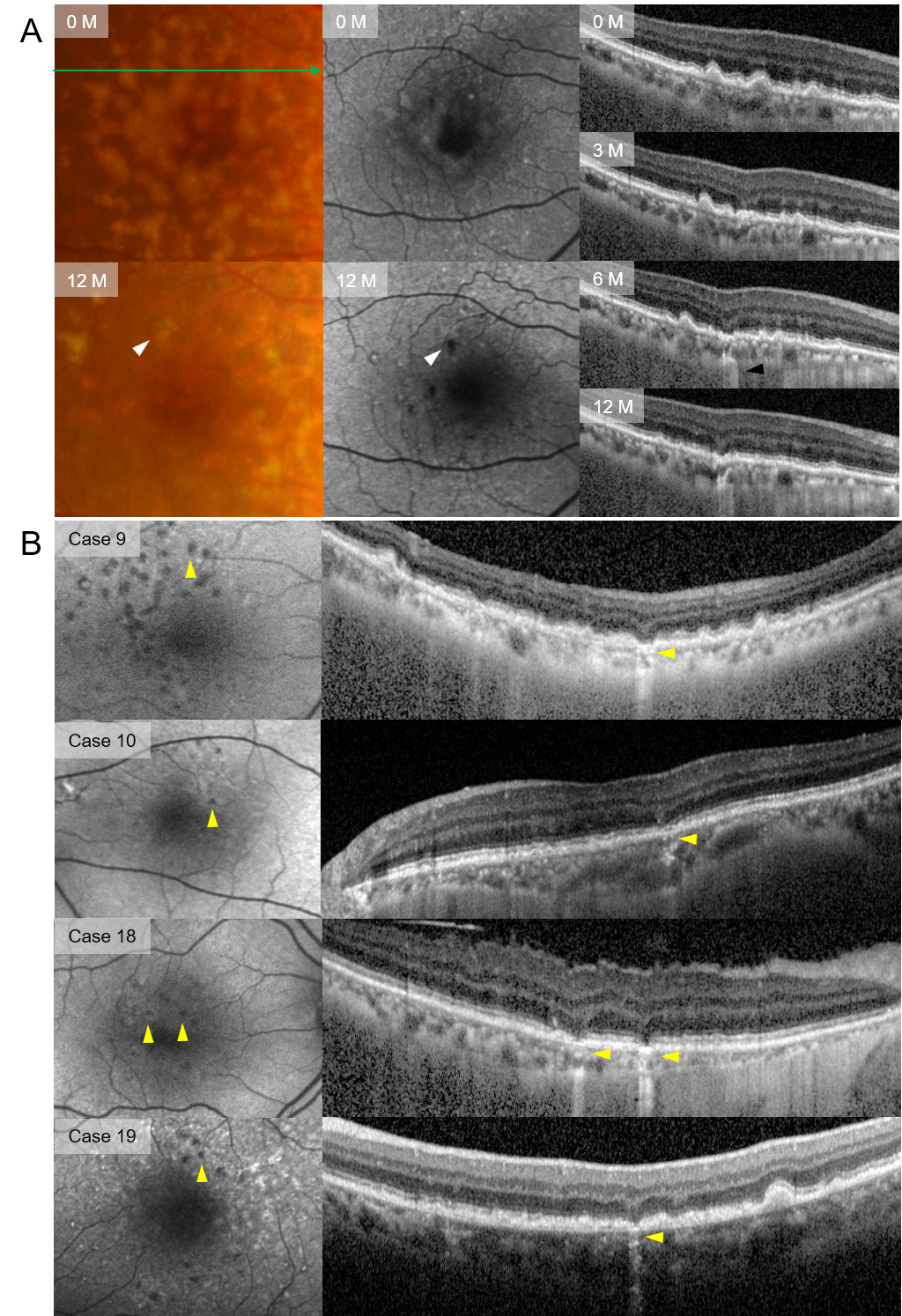


Supplementary Figure S4. (A) Serial color fundus photography and fundus autofluorescence (FAF) images of the study eye of case 4 at the initial examination and at 12 months, showing the regression of drusen with subsequent development of geographic atrophy (white arrowhead). Spectral-domain optical coherence tomography (SD-OCT) scans of the same point demonstrates the subsidence of the outer plexiform layer and inner nuclear layer at month 3, which is defined as nascent geographic atrophy. There is also increased signal transmission below the Bruch’s membrane at month 6 (black arrowhead). (B) Additional representative cases showing hypo-autofluorescent spots in FAF and corresponding SD-OCT images suspected of laser-induced atrophy (yellow arrowhead) at month 12.


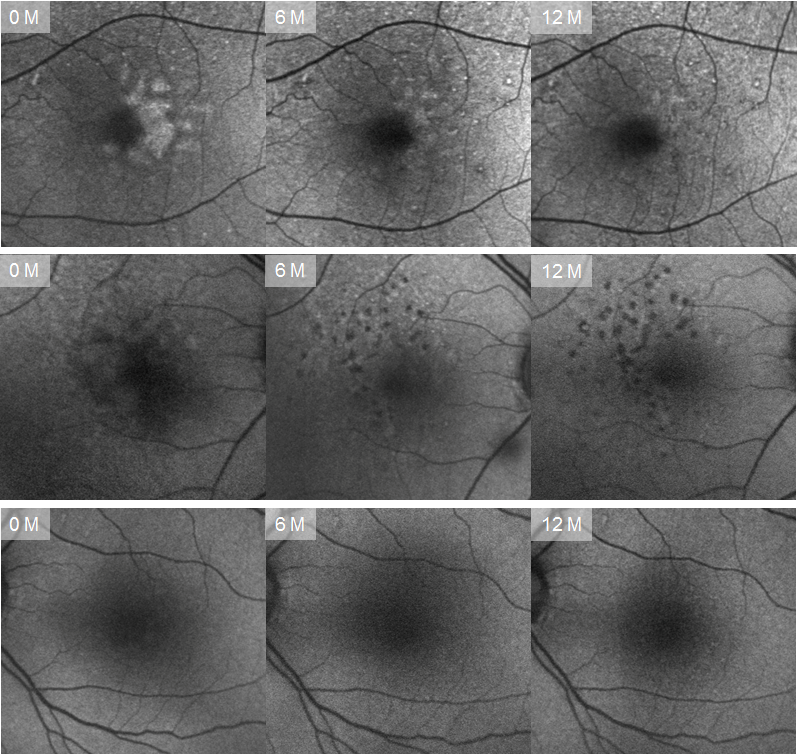


Supplementary Figure S5. On serial fundus autoflurescence (FAF) images of the study eyes, heterogenous patterns of autofluorescence were found over 12 months of follow up: 14 (70%) out of 20 study eyes showed focal hyper-autofluorescence at laser sites and gradually faded or changed into hypo-autofluorescence (6 cases) over 12 months. Two eyes (10%) showed focal hypo-autofluorescence and 4 eyes (20%) showed no gross change over 12 months of follow up on FAF.


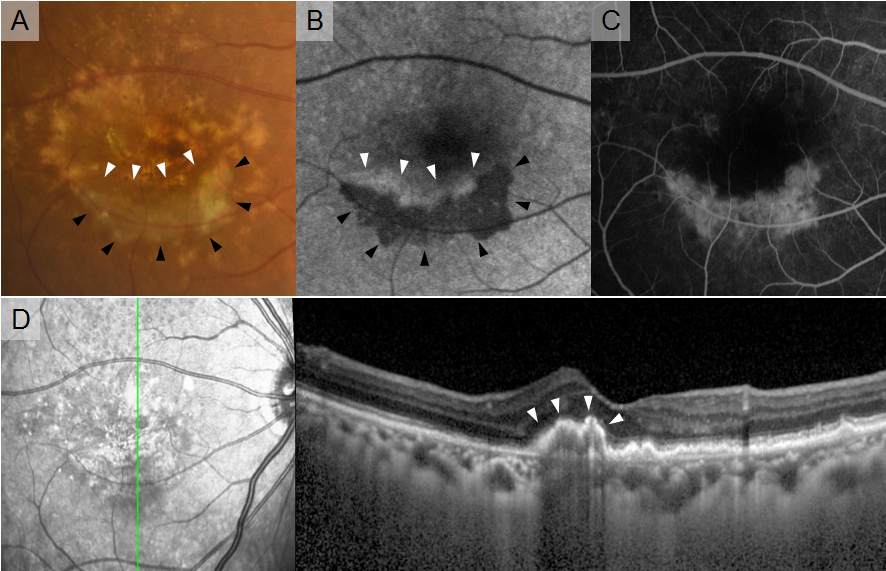


Supplementary Figure S6. Multimodal imaging of study eye with retinal pigment epithelium (RPE) tear at month 12 (Case 14). (A) Color fundus photography shows a depigmented area corresponding to the exposed choroid (black arrowheads) and superior hyperpigmented area corresponding to the scrolled RPE (white arrowheads). (B) Fundus autofluorescence image shows a delineated, hypoautofluorescent signal in the RPE tear area (black arrowheads) and a hyperautofluorescent area corresponding to the scrolled RPE (white arrowheads). (C) Fluorescein angiography reveals an early hyperfluorescence in the tear area with adjacent hypofluorescence due to blocks of rolled RPE. (D) In the vertical scan of spectral-domain optical coherence tomography, white arrow points an area of scrolled RPE causing choroidal backshadowing.


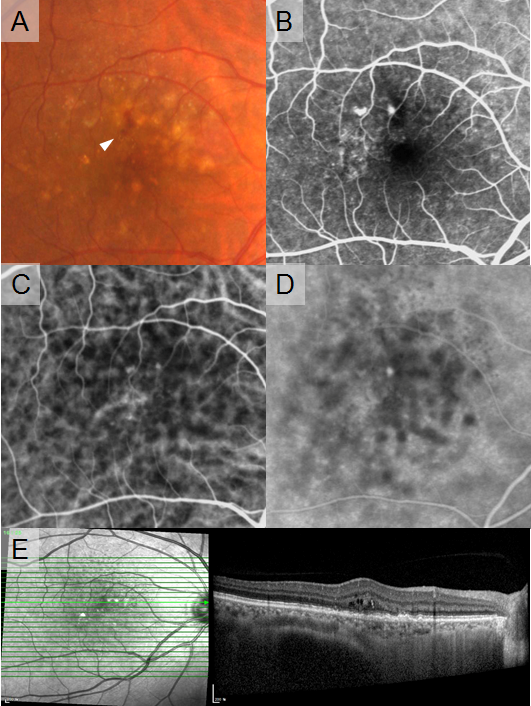


Supplementary Figure S7. Multimodal imaging of control eye with retinal angiomatous proliferation at month 6 (case 10). (A) Color fundus photography shows multiple drusen with small retinal hemorrhage (white arrowhead). (B) Fluorescein angiography reveals dense hyperfluorescent spots with diffuse leakage in adjacent area. (C) Magnification of indocyanine green angiography (ICGA) shows retinal-choroidal anastomosis. (D) Late-phase ICGA demonstrates a hot spot lesion. (E) Spectral-domain optical coherence tomography shows intraretinal cystoid spaces.


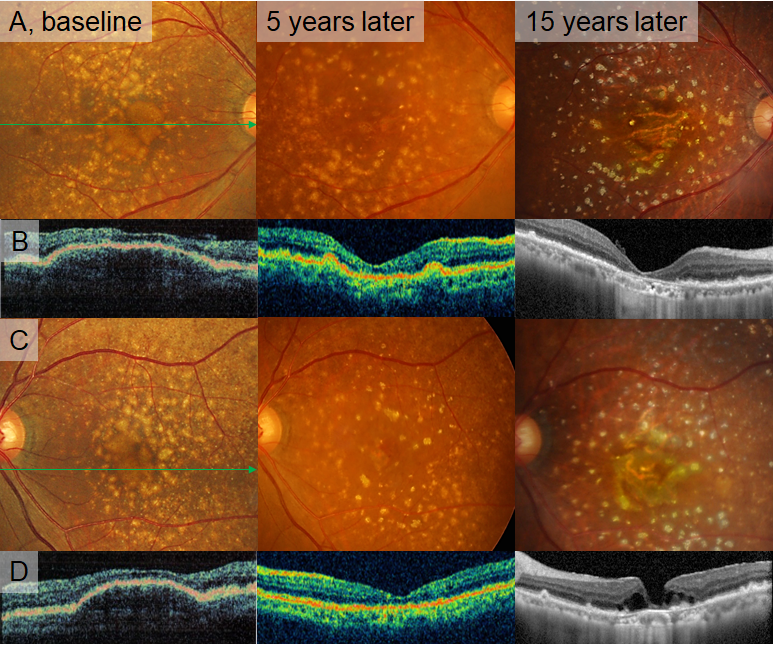


Supplementary Figure S8. A representative color fundus photography and spectral-domain optical coherence tomography (SD-OCT) images of 83-years-old female patients with drusenoid pigment epithelium detachment progressing to geographic atrophy over 15 years. Fundus photography and SD-OCT images of right eye (A, B) and left eye (C, D).
